# The genomic underpinnings of eukaryotic virus taxonomy: creating a sequence-based framework for family-level virus classification

**DOI:** 10.1186/s40168-018-0422-7

**Published:** 2018-02-20

**Authors:** Pakorn Aiewsakun, Peter Simmonds

**Affiliations:** Nuffield Department of Medicine, University of Oxford, Peter Medawar Building, South Parks Road, Oxford, OX1 3SY UK

**Keywords:** Virus, Metagenomic, Taxonomy, Virus classification, Taxon, Hidden Markov model, Baltimore classification

## Abstract

**Background:**

The International Committee on Taxonomy of Viruses (ICTV) classifies viruses into families, genera and species and provides a regulated system for their nomenclature that is universally used in virus descriptions. Virus taxonomic assignments have traditionally been based upon virus phenotypic properties such as host range, virion morphology and replication mechanisms, particularly at family level. However, gene sequence comparisons provide a clearer guide to their evolutionary relationships and provide the only information that may guide the incorporation of viruses detected in environmental (metagenomic) studies that lack any phenotypic data.

**Results:**

The current study sought to determine whether the existing virus taxonomy could be reproduced by examination of genetic relationships through the extraction of protein-coding gene signatures and genome organisational features. We found large-scale consistency between genetic relationships and taxonomic assignments for viruses of all genome configurations and genome sizes. The analysis pipeline that we have called ‘Genome Relationships Applied to Virus Taxonom**y**’ (GRAViTy) was highly effective at reproducing the current assignments of viruses at family level as well as inter-family groupings into orders. Its ability to correctly differentiate assigned viruses from unassigned viruses, and classify them into the correct taxonomic group, was evaluated by threefold cross-validation technique. This predicted family membership of eukaryotic viruses with close to 100% accuracy and specificity potentially enabling the algorithm to predict assignments for the vast corpus of metagenomic sequences consistently with ICTV taxonomy rules. In an evaluation run of GRAViTy, over one half (460/921) of (near)-complete genome sequences from several large published metagenomic eukaryotic virus datasets were assigned to 127 novel family-level groupings. If corroborated by other analysis methods, these would potentially more than double the number of eukaryotic virus families in the ICTV taxonomy.

**Conclusions:**

A rapid and objective means to explore metagenomic viral diversity and make informed recommendations for their assignments at each taxonomic layer is essential. GRAViTy provides one means to make rule-based assignments at family and order levels in a manner that preserves the integrity and underlying organisational principles of the current ICTV taxonomy framework. Such methods are increasingly required as the vast virosphere is explored.

**Electronic supplementary material:**

The online version of this article (10.1186/s40168-018-0422-7) contains supplementary material, which is available to authorized users.

## Background

Virus taxonomy is a man-made construct that seeks to describe and catalogue the vast diversity of known viruses and their genetic interrelationships. Viruses are formally classified into orders, families, genera and species by the International Committee on Taxonomy of Viruses (ICTV; https://talk.ictvonline.org/). This organisation maintains a universal taxonomy of viruses that encapsulates their extraordinary genetic and structural diversity. Viral diversity is far greater than encountered in other organisms, with major differences in their genetic material (RNA or DNA) and configurations (double or single stranded) and orientation of their encoded genes. Viral genomes may be segmented, often co-packaged together or, more frequently, into separate virions that are then required to productively infect a cell. Virion morphology and size varies from particles with icosahedral or more complex symmetries or may form filamentous, rectangular, bullet, even bottle-shaped nucleocapsids. Some viruses are enveloped in a host-derived lipid bilayer. Finally, viral genomes are hugely variable in size and their complements of genes, ranging from less than 2000 bases encoding 2 genes to 2.5 million base pairs encoding over 2500 genes [1].

So diverse are viruses in terms of their replication strategy and structure that viruses lack a common set of genes by which their deeper evolutionary relationships may be inferred. Unlike bacteria, fungi and other microorganisms, universal trees depicting their evolutionary histories cannot be constructed. Indeed, it is most likely that viruses may not share a common origin, but originate as parasitic companions of prokaryotes and eukaryotes at varying times in their host’s evolution.

The broadest division of viruses is the Baltimore classification, assignments that are based on their genome configurations as follows: I: dsDNA, II: ssDNA, III: dsRNA, IV: ssRNA sense orientation of genes, V: ssRNA, antisense orientation, VI: ssRNA with reverse transcription of a dsDNA replication intermediate and VII: dsDNA with a ssRNA replication intermediate [2]. With the exception of groups VI and VII, members of which show substantial similarities in genome organisation and replication strategies, this functional division splits viruses into groups that are largely or entirely unrelated to each other in evolutionary terms. However, the division is coarse with several groups, most evidently group I, containing several unlinked virus groups.

While current ICTV taxonomy has incorporated this diverse collection of evolutionarily related and unrelated groups into a single, overarching framework, there are further challenges from the explosion in virus nucleotide sequence data that have been accrued from next generation or high-throughput sequencing (HTS) methods. Their application to aquatic and terrestrial environmental samples, as well as to the gut microbiome, has revealed an astonishing diversity of virus sequences, many bacteriophages, but others likely infecting a range of eukaryotes, including amoebae, algae, insects, fish and plants [3–7]. The majority of such sequences do not match any of those of viruses in currently assigned taxa, and clearly, the ICTV classification would have to be greatly expanded to incorporate this much greater dataset of viruses.

Recently, the ICTV, on advice from an expert group [8], expressed the intention to consider the incorporation of viruses known only by their nucleotide sequences into the formal taxonomy. Classification of such viruses would be subject to there being coding complete genome sequences available and with appropriate quality control to ensure sequence accuracy and avoid problems of misassembly [8]. However, these newly described viruses lack information on their phenotypic properties that have historically been used in their classification, such as virion structure, pathogenicity in their hosts, replication mechanisms and epidemiology/transmission routes. It was therefore proposed that the genome sequence itself may be used to infer a number of properties that may be used as attributes that assist in their taxonomic assignments.

The policy to accept metagenomic-derived sequences into the ICTV taxonomy is not entirely new, and large numbers of recent assignments of further species and genera within existing families have been made in recent years [9]. Many such taxonomy additions, particularly at the level of species or genus, can be justified because there is an existing framework of taxon assignments within such families, often based upon phenotypic properties of isolates of their founder members.

However, the incorporation of viruses that are much more divergent from the existing virus datasets is far more problematic. The ICTV taxonomy provides little information that might guide decisions on the classification of more divergent viruses to existing families or conversely justifying the creation of new virus families or orders. Indeed, there is little or no systematic information on what genomic attributes delineate these higher taxonomic divisions; does simple possession of homologous genes or shared organisational features such as gene order and segmentation suffice to justify family assignment? Do genes encoding structural proteins and which therefore define virion morphology need to be shared? Is there any consistency in how viruses are currently divided into families and orders at the genomic level? These uncertainties require urgent resolution if further classification of the more divergent viruses discovered in recent HTS and related investigations are to proceed on a rational and consistent basis in the future.

In the current study, we have extracted genomic features recoverable from genome sequences of currently classified eukaryotic viruses and sought to determine which are best predictive of their family or order assignments in the most recent ICTV taxonomy. Eukaryotic viruses are the main focus of this study as their taxonomy is well established and populated, and can be used to validate a taxonomic assignment framework. Degrees of relatedness that underpin current family and order divisions were estimated by extraction from viral genome sequences both their organisational features (gene complements and gene orders), sharing of homologous genes and their amino acid sequence identity. Features in these multi-parameter datasets were evaluated for their ability to recover the taxonomy of all currently classified eukaryotic viruses in the ICTV Master Species List. The identification of informative genome features that can precisely recapitulate the current ICTV taxonomy allows classification of currently unassigned viruses from their sequences alone. This is a process we have termed ‘genome relationships applied to virus taxonomy (GRAViTy) assignments’, and its use may contribute to the foundation of a future, comprehensive, internally consistent sequence-only classification of viruses.

## Results

### Virus sequence and taxonomy information sources

A complete list of 3854 eukaryotic viruses for which complete genome sequences are available was assembled (Additional file 1: Table S1, Additional file 2: Table S2). These exemplify each of the current ICTV taxonomy assignments down to species level. This information was drawn from the ICTV Master Species List, the Virus Metadata Repository and further assignments approved by the ICTV Executive Committee in July 2017, currently under ratification vote. This collection provides the most complete and up-to-date collection of viruses with defined assignments.

### Relationship among viruses within each Baltimore classification group

The first step in the analysis was the extraction of information on those genomic features from complete genome sequences of each virus. This use of multiple features extracted from viral sequences as potential contributors to taxonomy assignments contrasts with traditional phylogenetic methods, in which viruses are often represented by only small, highly conserved portions of their genomes, such as the catalytic core of RNA-dependent RNA polymerase (RdRp) gene sequences for different groups of RNA viruses. Features extracted included gene complements, genomic organisation and metrics of gene homology. Herein, viruses are annotated with databases of protein profile hidden Markov models (PPHMMs) and genomic organisation models (GOMs). Instead of a molecular sequence, each virus is represented by a PPHMM signature and a GOM signature. A PPHMM signature is simply a list of the degrees of similarity of genes present in the virus to various PPHMMs in the database at the amino acid level. Similarly, a GOM signature is a list of the degrees of similarity of its genomic organisation to various GOMs in the database. Additional file 3: Table S3 summarises PPHMMs used in this study.

The second step was to estimate the degrees of virus relatedness through a multi-dimensional distance calculation based on a comparison of their PPHMM and GOM signatures. Sets of distances were transformed into a composite generalised Jaccard (CGJ) similarity index, *J*, which ranges in value between 0 (no detectable similarity) and 1 (sequence identity). Pairwise distances, *D*, simply 1 − *J*, were used to construct dendrograms with the unweighted pair group method with arithmetic mean (UPGMA) algorithm. This method circumvents the need to first identify and align homologous genes that are often highly divergent in sequence. The GRAViTy method, in contrast, allows dendrograms to be constructed across very divergent viruses within a Baltimore group (see the ‘Methods’ section) without prior intervention, modelling or evolutionary assumptions.

**Fig. 1:**
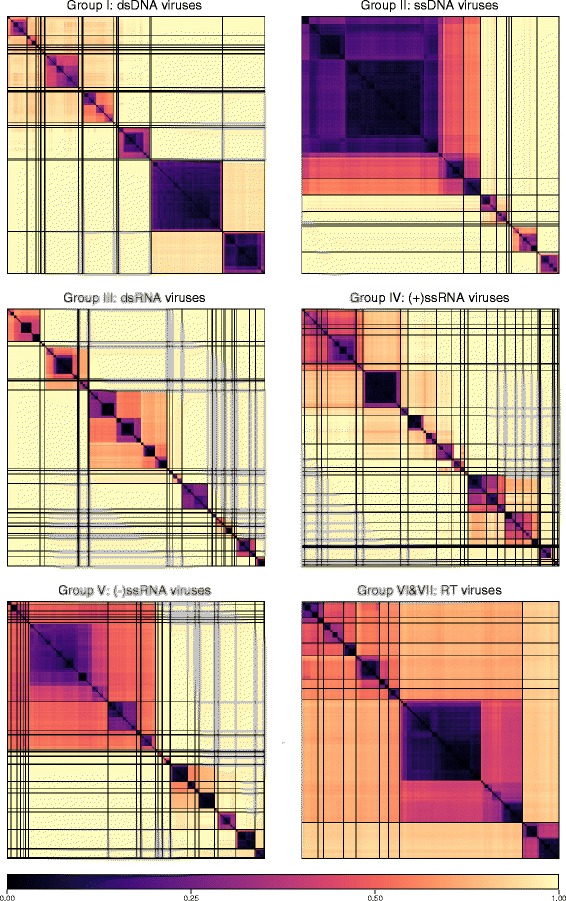
Heat maps of Jaccard distances between virus taxonomic groups. Pairwise distances, *D* (based upon 1 – *J*, the composite generalised Jaccard similarity index) were computed between each sequence in the Baltimore group and plotted on heat maps as colour-coded points (see scale at the bottom of the figure). The light grey solid lines indicate boundaries between each virus taxonomic group, and the data is organised such that groups with high similarities are closer to one another. For larger heat maps with annotations for virus family and order, see Additional file9: Figures S1–S6

**Fig. 2:**
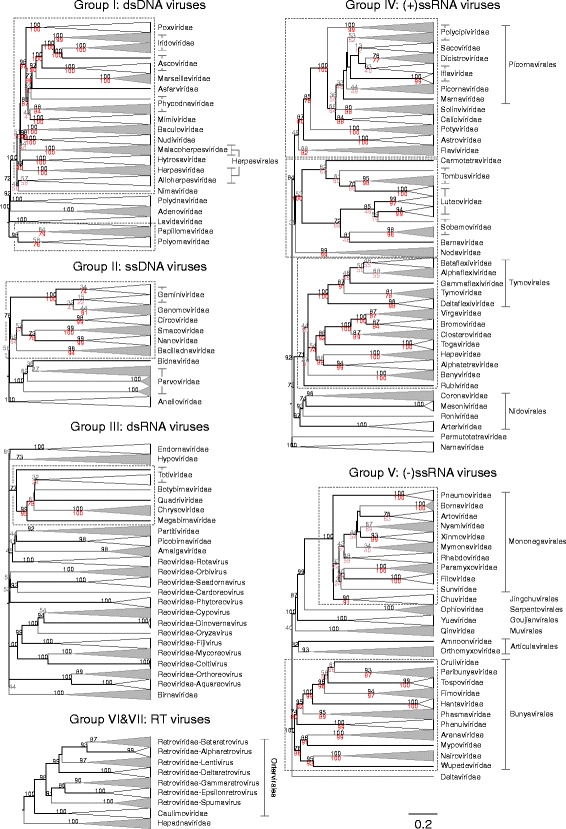
Virus dendrograms based on composite Jaccard distances. UPGMA dendrograms were constructed from pairwise distance matrices shown in Fig. 1. Tips are labelled with family and genus assignments used in our virus classification study. Virus taxonomy at the order level is also shown to the right of the dendrograms. The scale bar for *D* is shown at the bottom (see Additional file 10: Figures S7–S12 for dendrograms additionally annotated for individual sequences (accession numbers) and genus assignments). Bootstrap clade support values (≥ 30%) are shown on the branches. Those in black (≥ 70%) and grey (< 70%) were calculated for the entire dendrograms. A number of specific clades were re-bootstrapped (dotted boxes) with pruned signature tables, and for these, the derived clade support values are shown in red (≥ 70%) or pink (< 70%)

Sets of pairwise distances between members within each Baltimore group were visualised through colour-indexed heat maps (Fig. 1; Additional file 9: Figures S1–S6). Phylogeny relations between individual group and viral sequences were determined through construction of a UPGMA dendrograms (Fig. 2; Additional file 10: Figures S7–S12). All dendrograms preserved the pairwise distances well (cophenetic correlation between the dendrogram and the distance matrix of group I = 0.994, group II = 0.984, group III = 0.995, group IV = 0.964, group V = 0.993, groups VI and VII = 0.990), indicating that the two are consistent.

Overall, there was a close concordance between sequence groupings and their current ICTV assignments into families. The relationships are all the more remarkable for being based on distance metrics constructed from sets of genomic attributes without any pre-selection for what might be considered to be more informative metrics. The analysis reveals a primary division of viruses at the family level that, with very few exceptions, were readily identifiable as tight clusters with ≥ 70% bootstrap support. Family-specific groupings possessed relatively long branch lengths and separation from other clades (Fig. 2; Additional file 10: Figures S7–S12) and, more impressionistically, were typically visualised as squares of intense colour in a background of yellow (Fig. 1; Additional file 9: Figures S1–S6).

Further detailed examination of dendrograms constructed from CGJ distances revealed, however, a small number of instances of families not being monophyletic, including separation of rubella virus (genus *Rubivirus*) from the rest of togaviruses, and polyphyletic groupings of reoviruses. A detailed analysis of these exceptions and the extent to which other analyses support the GRAViTy groupings or the current ICTV classification is provided in Additional files 11 and 13. Summarising, most differences in grouping between GRAViTy analysis of these virus groups and their current taxonomy have been reported previously and are consistent with virus relationships determined by other methods such as genome phylogenies (Additional file 11: Figures S13–S15). For most, CGJ distances provide further evidence to support their future taxonomic reassignments.

Virus taxonomy at the level of order could also be recovered with members in different families generally having much lower and distinct CGJ similarity scores than those between members of the same family. GRAViTy relationships that recapitulated currently assigned orders included *Tymo*-, *Nido*- and *Bunyavirales* with 100, 88 and 100% bootstrap support, respectively. Further supported grouping at the level of order included the recently proposed *Jingch*-, *Serpento*-, *Goujian*-, *Mu*-, *Aricula*- and *Ortervirales* (Fig. 2). Although not formally assigned as an order, the evolutionarily related nucleo-cytoplasmic large DNA viruses (NCLDV; [10]), including *Asco*-, *Irido*-, *Asfar*-, *Marseille*-, *Phycodna*-, *Pox*- and *Mimiviridae* families, formed a separate grouping from other large DNA viruses, with 98% bootstrap support.

*Picorna*-, *Mononega*- and *Herpesvirales* were however not monophyletic. For *Picornavcirales*, members of the *Caliciviridae* and *Solinviviridae* families, which are not classified into this order, were embedded within the clade, while *Potyviridae* showed a sister relationship. However, this phylogeny is indeed consistent with the previously noted relationships of these groups based on RdRp phylogenies and originates from discrepancies in replication gene relationships from structural protein structures that define their capsid morphology and symmetry [11].

*Chuviridae*, the sole family of the order *Jingchuvirales*, was positioned within the clade of *Mononegavirales* and separated *Pneumoviridae* from the rest of the order, although the branch separating them was short and not bootstrap supported (Fig. 2; Additional file 10: Figure S9). Collectively, however, the two orders form a monophyletic clade with 100% bootstrap support.

The inclusion of the three families (*Herpes*-, *Alloherpes*- and *Malacoherpesviridae*) in the order *Herpesvirales* is primarily based upon their characteristic capsid morphology, without readily detectable sequence homology that defines this order [12]. We found that the only shared profile across these three families was between their genes coding for DNA packaging terminase, consistent with previous analyses [13]. Nevertheless, the herpesvirus families were collectively embedded within a larger clade of large DNA virus which exhibit detectable, similarity to each other through homologous DNA polymerase, protein kinase and ribonucleotide reductase genes (*Baculo*-, *Nudi*-, *Hytrosa*-, *Asco*-, *Irido*-, *Asfar*-, *Marseille*-, *Phycodna*-, *Pox*-, *Mimi*- and *Nimaviridae*). This higher level grouping showed 100% bootstrap support but excluded the *Polydnaviridae* that showed a much less degree of relatedness to other large DNA viruses (74% bootstrap support). For this latter virus family, its two genera are considered to be independently derived from perhaps an ancestral nudivirus (*Bracovirus*) and another large cytoplasmic DNA virus (*Ichnovirus*) [14]. They nevertheless formed a bootstrap supported but highly divergent clade, reflecting shared profiles of their cysteine-rich protein-coding genes (c4.1 and d9.2 of the *Hyposoter fugitivus* ichnovirus, homologues in Campoletis sonorensis ichnovirus and CRP1 and CRP3 proteins of the Cotesia congregate brachovirus [15–17]). Finally, there was further support for all DNA viruses possessing DNA polymerase creating a larger clade that encompassed *Adenoviridae* and *Lavidaviridae* from which the small DNA virus families of *Polyomavirdae* and *Papillomaviridae* were excluded (100% bootstrap support). These two latter families were, however, linked through PPHMM matches of their E1 and NS proteins corresponding to the previously noted protein sequence homology [18, 19], creating a relatively deeply branching clade with 98% bootstrap support.

In group II, *Gemini*-, *Genomo-*, *Circo*-, *Smaco*-, *Nano*- and *Bailladnaviridae* families, clustered together through possession of the *rep* gene (78% bootstrap support), corresponding to membership of the circular replication-associated protein encoding single-stranded (CRESS) group of ssDNA viruses [20, 21] while *Parvoviridae* and *Anelloviridae* fell into separate groups. Among the dsRNA viruses in group III, we observed unrecognised inter-family relationships included groupings of *Chryso*-, *Quadri*-, *Megabirna*-, *Botybirna*- and *Totiviridae* (77% bootstrap support). There was a similar grouping between *Bromo*-, *Virga*-, *Clostero*- and *Togaviridae* of group IV (88% bootstrap support), a grouping partly congruent with the previously proposed ‘alpha-like’ group of viruses [22]. This clade in turn grouped within a larger clade with 71% bootstrap support that included *Alphatetraviridae*, *Hepeviridae*, *Benyviridae, Rubivirus*, the four established families within the *Tymovirales* (*Alpha-*, *Beta-* and *Gammaflexiviridae* and *Tymoviridae*) and *Deltaflexiviridae* (a recently proposed new family within the order *Tymovirales*). Lists of protein profiles connecting these families together, responsible for these higher virus taxonomical structures, can be found in Additional file 4: Table S4.

### Methods for bootstrap resampling

While all classified virus families formed distinct clusters in the dendrograms and heat maps, a minority of families (*n* = 10) showed bootstrap support below 70%, a commonly accepted measure indicative of the robustness of groupings. These comprised the *Papilloma*- (64% bootstrap support), and *Polyomaviridae* (58% bootstrap support) in Baltimore group I, *Gemini*- (34/15% bootstrap support), and *Genomoviridae* (44%) in group II, *Totiviridae* (32%) in group III, and *Picornaviridae* (44%) and several other families in group IV, and finally, *Mymona-* (34%) and *Rhabdoviridae* (48%) in group V (Fig. 2). Apart from simply representing a more diverse group of viruses, the lower bootstrap support values for some virus families may have originated from possession of mosaic genomes and conflicting PPHMM and GOM signature relationships with other viruses. Another contributor may have arisen through the nature of bootstrapping resampling of a relatively sparse table of PPHMM and GOM signatures and the failure to sample the intended number of entries, or in extreme cases, any at all.

To investigate this, we separated the dataset into sub-groups that shared no signatures between them. Examples included the separation of *Polyomavirdae* and *Papillomaviridae* from other DNA viruses that shared PPHMM signatures associated with DNA polymerase and other replicative geners. We then reformulated separate signature tables for the two groups to exclude PPHMMs and GOMs that were not relevant to members of each (see the ‘Methods’ section for details). This substantially reduced the bootstrap sampling space, particularly for *Polyomavirdae* and *Papillomaviridae*, that possess relatively small genomes compared to other dsDNA viruses. Using these modified tables, bootstrap support did increase substantially for *Papilloma*- (64 to 79% bootstrap support, Fig. 2, red labelling), and *Polyomaviridae* (58 to 76%). Similar improvements in bootstrap support were noted in several further virus families in other Baltimore groups including *Geminiviridae* (74%), *Genomoviridae* (61%) and *Flaviviridae* (82%). Nevertheless, bootstrap support for many other families remained relatively unchanged, suggesting other underlying causes for their less robust groupings (see the ‘Discussion’ section).

### Informative genome features that group viruses according to ICTV taxonomy

**Fig. 3:**
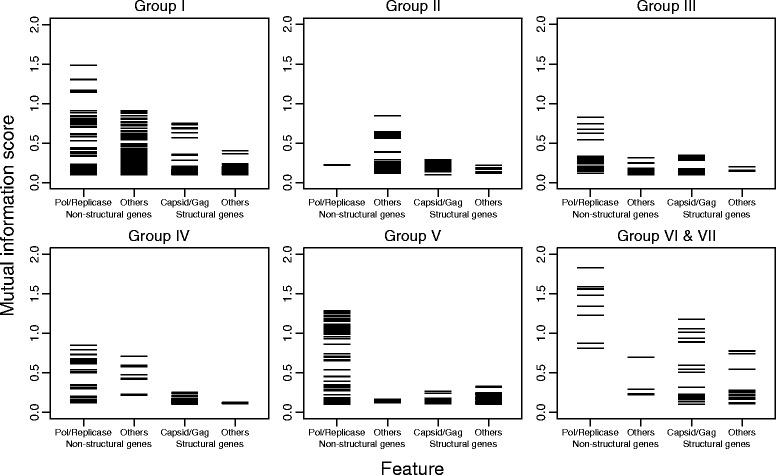
Feature importance in different virus groups for family assignments. Mutual information (MI) scores are used to evaluate what features were predictive of virus taxonomy. Features with high MI scores are those that vary among virus taxonomic groups, but are at the same time, shared values by viruses in the same family. Only features associated with protein profiles and have MI scores greater than 0.1 are shown. Assignments to replicative, other non-structural and structural genes are described in the ‘Methods’ section

Mutual information (MI) scores were calculated to evaluate what genes were predictive of virus taxonomic relationships. The greater the value, the higher the dependency between the feature and the taxonomic assignment (Fig. 3; Additional file 5: Table S5). In general, PPHMMs of genes involved in replication, particularly those encoding polymerases, showed the highest MI scores. There was a sharp drop in MI scores between the features associated with non-structural proteins and with structural proteins (Fig. 3). This result reflects the generally greater sequence conservation of many replication-related proteins of eukaryotic viruses within the same families but, at the same time, indicates that they are different enough among viruses of different families to be useful for virus classification purpose.

### Taxonomic assignment framework and cross-validation

**Fig. 4:**
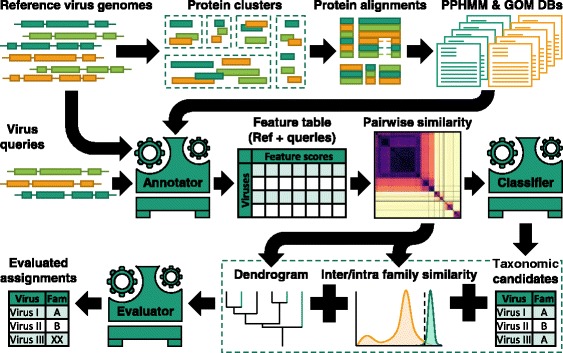
Overview of virus taxonomy prediction by GRAViTy. Schematic diagram of the processing steps used to construct classifiers based on viruses with assigned taxonomic status (reference virus genomes) and the pipeline used to classify viruses of interest (virus queries). In summary, protein sequences are extracted from reference virus genomes and clustered based on pairwise BLASTp bit scores. Sequences in each cluster are then aligned and turned into a protein profile hidden Markov model (PPHMM). Reference genomes are subsequently scanned against the database of PPHMMs to determine the locations of their genes and genomic organisation models (GOMs) for each virus family are constructed. PPHMM and GOM databases are the main machinery of our genome annotator (Annotator). To classify viruses of interest, they, together with the reference viruses, are first annotated with information on the presence of genes and the degree of similarity of their genomic organisation to various reference families (Feature table). Pairwise similarity scores (composite generalised Jaccard similarity) is then estimated and passed to the classifier to identify taxonomic candidates for each query using the 1-nearest neighbour algorithm. A UPGMA dendrogram and a similarity acceptance cut-off for each virus family are also estimated from the pairwise similarity scores and used by the evaluator to evaluate the taxonomic candidates. The analysis is performed in parallel for the six virus Baltimore groups; those showing best matches are the finalised taxonomic assignments

To investigate the ability of the framework to correctly assign known and unknown viruses, sequences from the classified dataset were sampled and analysed through six sub-pipelines, one for each Baltimore group. Each possesses three compartments: (i) feature annotator—annotates viruses of interest with databases of PPHMMs and GOMs; (ii) classifier—assigns taxonomic groups to sequences based on their genome annotation; and (iii) classification evaluator—decides whether or not to accept or reject the taxonomic proposals (Fig. 4). Briefly, in each sub-pipeline, feature annotator produces PPHMM and GOM signatures for virus queries. Pairwise similarities between the signatures of the queries and those of viruses in the database are then computed. The classifier in turn proposes taxonomic groups to viruses according to their most similar counterparts in the database (1-nearest neighbour). The taxonomic proposals were then either accepted or rejected depending on the immediate neighbourhood of the viruses in the dendrogram.

The sensitivity and specificity of the method—its ability to correctly differentiate assigned viruses from unassigned viruses, and classify them into correct taxonomic group—was evaluated by threefold cross-validation technique. In this analysis, each virus taxonomic group was randomly divided into a reference set (67%) and a test set (33% of sequences). Groups with less than three samples were always put into the test set. Using the known taxonomy assignments of viruses in the reference set, the classifier was used to predict the taxonomy assignments of viruses in the test set. Its ability to do this was evaluated as (a) *sensitivity*, the ability of the classifier to recognise and correctly assign viruses in the test set and (b) *specificity*, its ability to correctly recognise viruses in the test dataset as unclassified when their taxonomic groups are not represented in the reference set. These metrics were separately evaluated for each sub-pipeline and for each of the threefold samplings. Overall, the sensitivity and specificity of the classifier ranged from 95.7 to 100% (mean 99.1%) for sensitivity and 99.3 to 100% (mean 99.8%) for specificity (Table 1; Additional file 6: Table S6). These results indicate that the genome features and distance metrics we have developed were both extremely sensitive and accurate. It therefore may guide genome only based classification of viral sequences in wider metagenomic datasets in a way that is consistent with current ICTV taxonomy.

**Table 1:** Performance of GRAViTy as evaluated by threefold cross-validation analysis

Sub-pipeline		‘Known’ viruses^1^							‘Unknown’ virus^2^				
		*n*	Assigned to the correct group		Assigned to a wrong group		Assigned as ‘unknown’		*n*	Assigned as ‘unknown’		Assigned to an existing group	
Group I: dsDNA virus	CV1	192	189	98.44%	0	0.00%	3	1.56%	1117	1117	100.00%	0	0.00%
	CV2	194	188	96.91%	1	0.52%	5	2.58%	1117	1117	100.00%	0	0.00%
	CV3	192	190	98.96%	0	0.00%	2	1.04%	1124	1124	100.00%	0	0.00%
	Overall	–	–	98.10%	–	0.17%	–	1.73%	–	–	100.00%	–	0.00%
Group II: ssDNA virus	CV1	369	369	100.00%	0	0.00%	0	0.00%	940	939	99.89%	1	0.11%
	CV2	371	369	99.46%	0	0.00%	2	0.54%	940	939	99.89%	1	0.11%
	CV3	370	370	100.00%	0	0.00%	0	0.00%	946	945	99.89%	1	0.11%
	Overall	–	–	99.82%	–	0.00%	–	0.18%	–	–	99.89%	–	0.11%
Group III: dsRNA virus	CV1	69	68	98.55%	0	0.00%	1	1.45%	1240	1233	99.44%	7	0.56%
	CV2	70	67	95.71%	0	0.00%	3	4.29%	1241	1232	99.27%	9	0.73%
	CV3	69	67	97.10%	0	0.00%	2	2.90%	1247	1239	99.36%	8	0.64%
	Overall	–	–	97.12%	–	0.00%	–	2.88%	–	–	99.36%	–	0.64%
Group IV: (+)ssRNA virus	CV1	415	415	100.00%	0	0.00%	0	0.00%	894	891	99.66%	3	0.34%
	CV2	412	411	99.76%	1	0.24%	0	0.00%	899	897	99.78%	2	0.22%
	CV3	415	412	99.28%	1	0.24%	2	0.48%	901	896	99.45%	5	0.55%
	Overall	–	–	99.68%	–	0.16%	–	0.16%	–	–	99.63%	–	0.37%
Group V: (−)ssRNA virus	CV1	176	176	100.00%	0	0.00%	0	0.00%	1133	1130	99.74%	3	0.26%
	CV2	177	177	100.00%	0	0.00%	0	0.00%	1134	1132	99.82%	2	0.18%
	CV3	180	179	99.44%	0	0.00%	1	0.56%	1136	1135	99.91%	1	0.09%
	Overall	–	–	99.81%	–	0.00%	–	0.19%	–	–	99.82%	–	0.18%
Groups VI and VII: RT virus	CV1	47	47	100.00%	0	0.00%	0	0.00%	1262	1262	100.00%	0	0.00%
	CV2	46	46	100.00%	0	0.00%	0	0.00%	1265	1265	100.00%	0	0.00%
	CV3	49	49	100.00%	0	0.00%	0	0.00%	1267	1267	100.00%	0	0.00%
	Overall	–	–	100.00%	–	0.00%	–	0.00%	–	–	100.00%	–	0.00%
Overall		–	–	99.09%	–	0.06%	–	0.86%	–	–	99.78%	–	0.22%

^1^Known in the sense that members of the family were in the reference dataset and that viruses in the same family in the test dataset should be classifiable

^2^Unknown in the sense that no members of the family were in the reference dataset, and therefore, viruses of that family in the test dataset should not be assigned

### Taxonomy relationships of currently unclassified viral gene sequences

GRAViTy was used to explore the diversity and potential future taxonomy assignments of real-world sequences described in recently published virus sequence datasets [23–28]. These include a wide range of small DNA virus and RNA virus sequences from environmental samples and from arthropods whose virome is known to be highly diverse but currently poorly characterised (Additional file 7: Table S7).

**Fig. 5:**
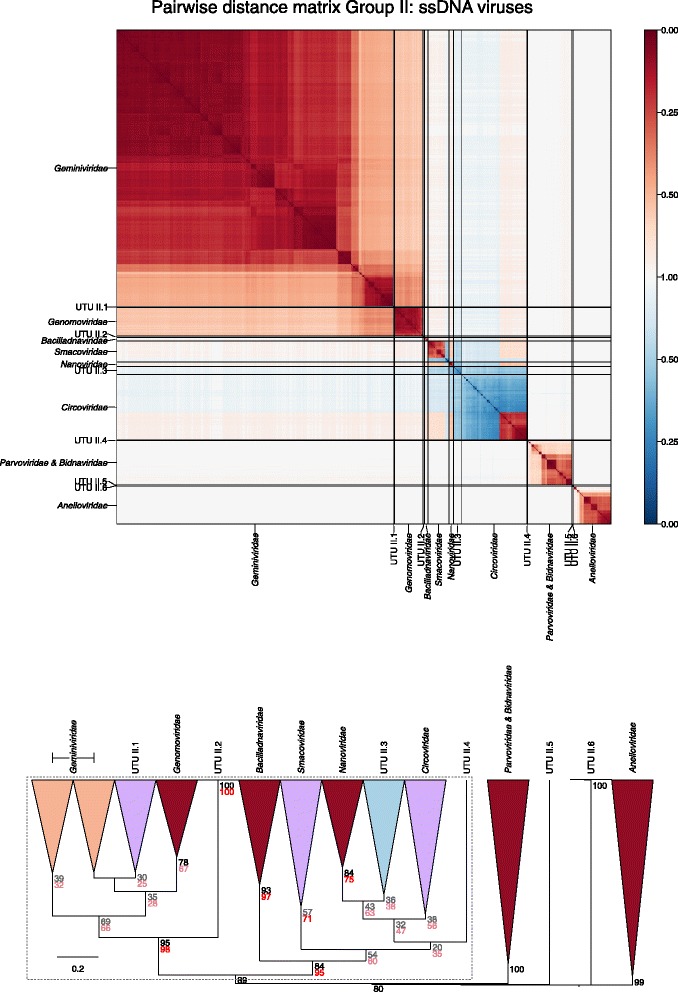
Genome relationships of metagenomic-derived viruses in Baltimore group II. Pairwise distance matrices (upper panel) and dendrogram (lower panel) for ssDNA viruses classified by ICTV (red) and newly described, currently unclassified viruses (blue). Novel taxa predicted by GRAViTy are labelled as unassigned taxonomy units (UTU) and numbered sequentially. Bootstrap clade support values (≥ 30%) are shown on the branches. Those in black (≥ 70%) and grey (< 70%) were calculated for the entire dendrograms. Several clades were re-bootstrapped with pruned signature tables (dotted boxes), and the re-bootstrap clade support values are shown in red (≥ 70%) or pink (< 70%). The shading of clades depicts the degree of bootstrap support; ≥ 70% dark shading; < 70% light shading. Clades containing both classified and unclassified viruses were shaded in purple

Viruses characterised as possessing ssDNA circular genomes [23–26] showed detectable similarity only to viruses in the Baltimore group II dataset. Through CGJ distance measurements and positions in the dendrograms, several fell into genetically divergent clades within the broader group of *rep*-containing viruses. These groups termed ‘unassigned taxonomy units’ (labelled as UTU II.1–UTU II.4; Fig. 5; Table 2; Additional file 12: Figure S16) were bootstrap supported and represent potentially additional virus families should these relationships be corroborated by further analysis, such as *rep*-gene phylogeny and other comparative genomic metrics. Other viruses expanded the diversity of the existing assigned virus families *Circoviridae* and *Smacoviridae*.

**Table 2:** Taxonomic groups predicted by GRAViTy in metagenomic datasets

Group	Known families^1^	Total assigned	Unclassified^2^	Total assigned
II	2	106	6	31
III	9	51	4	19
IV	23	703	101	388
V	9	61	16	22
Totals	43	921	127	460

^1^Number of existing families into which metagenomic sequences fell (total numbers of metagenomic sequences assigned to these are listed in column 3)

^2^Number of taxonomic groupings separate from classified virus families that may be assigned family status

**Fig. 6:**
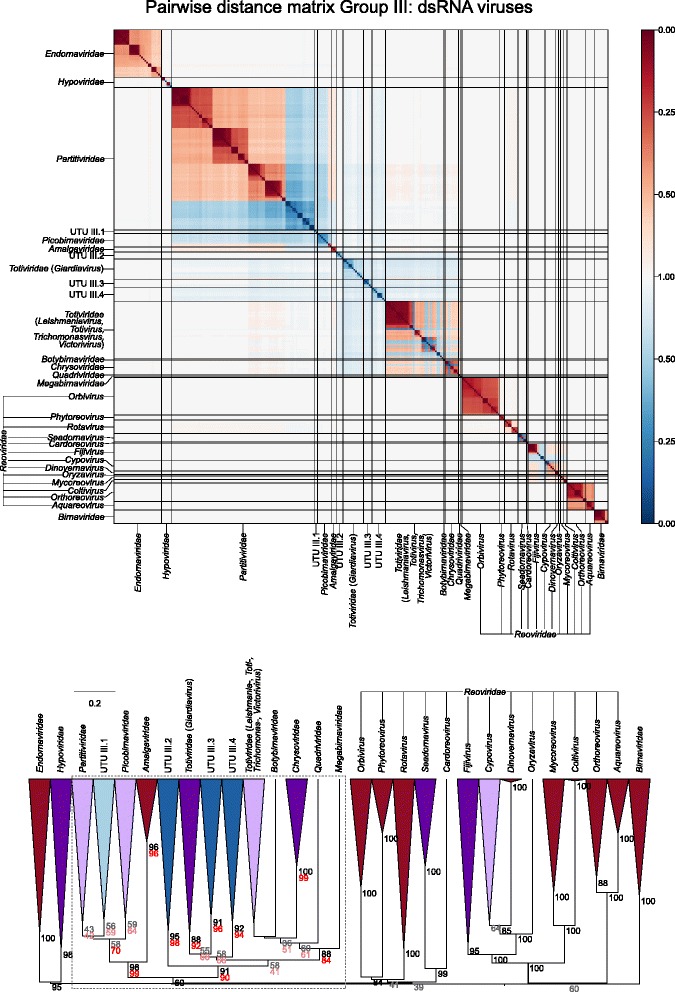
Genome relationships of metagenomic-derived viruses in Baltimore group III (see legend to Fig. 5)

There was a similar mix of novel groups and further examples of existing families on analysis of unclassified RNA viruses linked to Baltimore group III (Fig. 6; Table 2; Additional file 12: Figure S17). Examples of the former included a series of clusters (UTU III.3 and UTU III.4) most similar to, but grouping separately from, *Totiviridae* and the *Giardiavirus* genus sequences. There were several further examples of *Totiviridae*, *Partitiviridae* (forming two new within-family groups) and *Hypoviridae* and viruses related to the *Fijivirus* and *Seadornavirus* genera within separate branches of the *Reoviridae*. Finally, there were potentially as many as four new families of presumed dsRNA viruses in the data set.

**Fig. 7:**
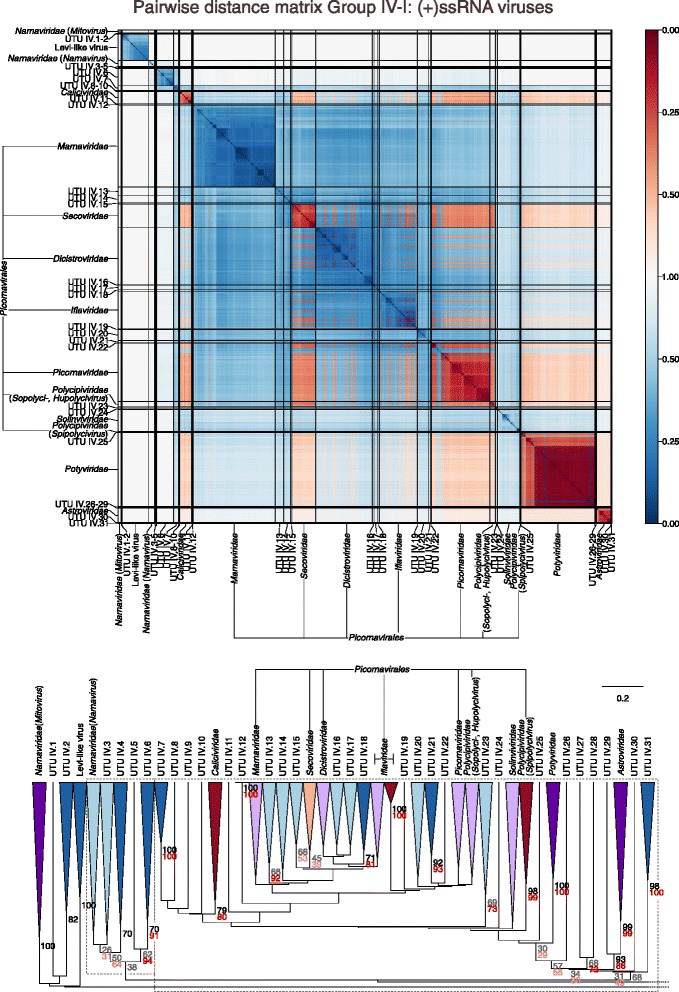
Genome relationships of metagenomic-derived viruses in Baltimore group IV, part 1 (see legend to Fig. 5)

**Fig. 8:**
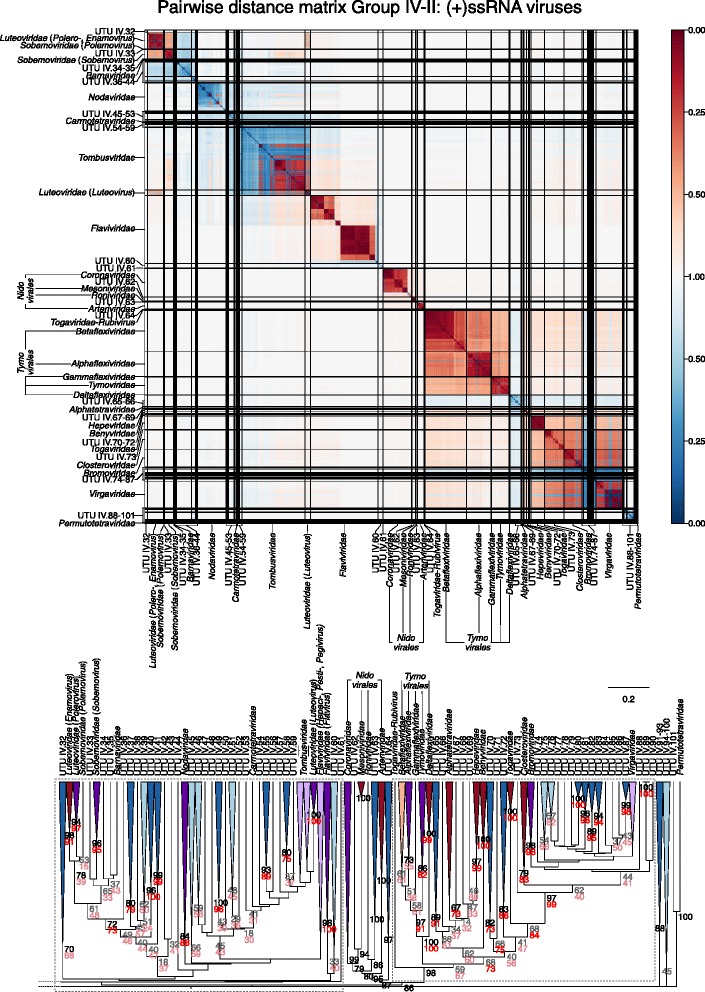
Genome relationships of metagenomic-derived viruses in Baltimore group IV, part 2 (see legend to Fig. 5)

A very large number of newly described viruses grouped with members of the *Picornavirales* and related viruses and their addition both expanded this order and changed inter-family relationships of currently classified viruses within it (Fig. 7; Table 2; Additional file 12: Figure S18). As described above, both GRAViTy and RdRp phylogenies did not fully resolve existing viruses into the families to which they are assigned (*Iflaviridae*, *Polycipriviridae* and *Picornaviridae*), and the addition of further metagenomically derived viruses created several new groups and additions to existing families, often though at the expense of decreasing the resolution of their groupings (Fig. 7, lower panel). Elsewhere, a remarkable number of new groupings might be assigned family status on further analysis (UTU IV.1–UTU IV.101; Figs. 7 and 8; Additional file 12: Figure S18; Additional file 7: Table S7).

**Fig. 9:**
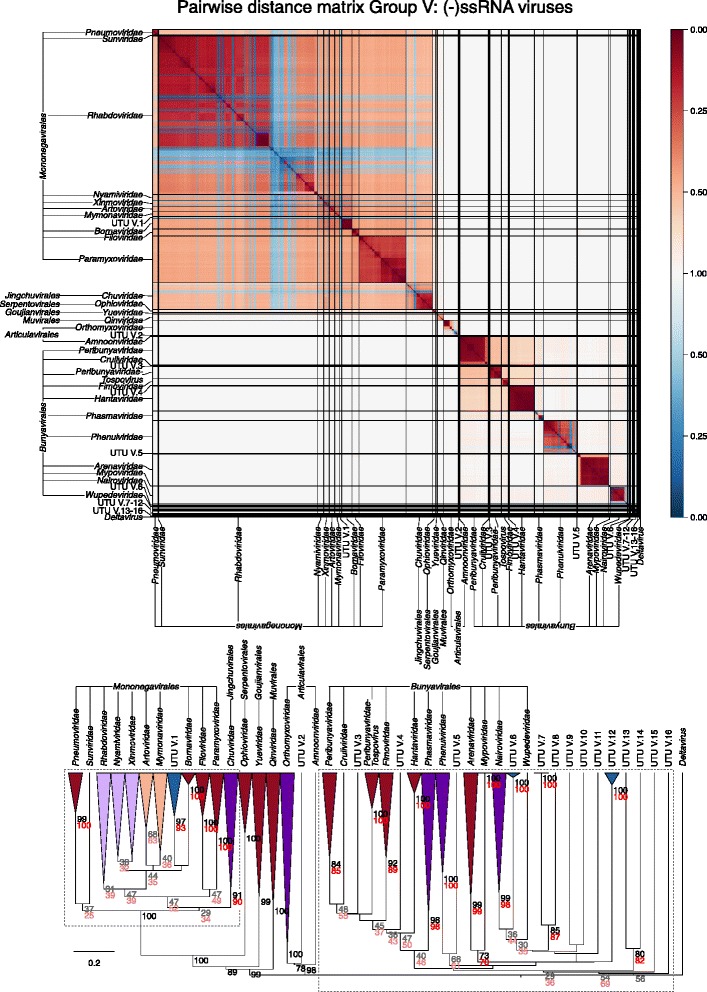
Genome relationships of metagenomic-derived viruses in Baltimore group V (see legend to Fig. 5)

The metagenomic datasets further contained over 80 genome sequences showing closest links to group V (Fig. 9; Table 2; Additional file 12: Figure S19) and represent presumed negative-stranded RNA viruses. Of these, a large number grouped with species of *Rhabdoviridae* (Fig. 9) while others formed a total of 16 separate UTUs with variable bootstrap support (Fig. 9, lower panel).

## Discussion

### Virus classification methods

GRAViTy provides the means to perform large-scale, multiple sequence alignment-free analysis of genetic relationships between virus datasets. The analysis presented in the current study (Figs. 1 and 2; Additional files 9 and 10: Figures S1–S12) provides evidence for large-scale consistency between genomic features and existing taxonomy assignments across Baltimore groups and between viruses ranging in size from 2700 bases to > 2.5 million bps. It can additionally reliably identify and assign known viruses to their correct families and reliably not assign viruses that are not in the training set, analogous to novel viruses (Table 1). The close linkage between traditional family assignments and their genomic features can therefore be exploited at least as an initial guide to the classification of larger datasets of viral sequences obtained from metagenomic studies using their sequences alone. Furthermore, because it is operating in the same overall framework as that reproduced the existing classification of eukaryotic viruses, its predictions can be represented as an extension of the ICTV taxonomy by the same assignment rules. It is, in effect, expanding what was originally a disease- and virion morphology-based classification of viruses into one that can incorporate viruses where such phenotypic information may never be obtained.

The use of PPHMMs for all coding sequences within a viral genome and collection of other information on its organisation to construct metrics of relatedness provides a holistic assessment of overall virus relatedness. It therefore avoids, at least in part, potentially unrepresentative assignments of viruses with mosaic genomes in which individual gene phylogenies may be misleading. It further avoids the need for pre-alignment of, often highly divergent, (multiple) sequences to construct evolutionary trees or calculate pairwise distances—these require making many assumptions and are potentially distorted through the inclusion of often non-homologous gene regions. The use of whole genome data by GRAViTy similarly avoids the need to pre-suppose which individual genes are informative for classification and which ones are not. There is the additional practical issue that, while GRAViTy can rapidly establish similarity metrics across an entire virus reference dataset, the alternative of multiple sequence alignment-based evolutionary reconstructions is intrinsically subjective in gene and regions selected and produces results that can be problematic to interpret on an automated platform. The requirement of GRAViTy for (near-) complete genome sequences has the converse effect of restricting its use for virus assignments where such coverage is available. This highlights the difference between virus *assignments*, in which new taxa may be characterised and assigned genomically using methods such as GRAViTy, and virus *identification*. For the latter, high-throughput methods such as BLASTn or BLASTx are more suitable large-scale virus identification of shorter, unassembled sequences that might typically be present in raw sequence data in the metagenomic dataset.

As a final methodological point, virus relationships in the current study have been displayed as dendrograms and as heat maps, in which the multi-parameter relationships between viruses are condensed into a single CGJ distance. However, these data presentations are a simplification of actual virus relationships since equivalent CGJ distances between one sequence and its neighbours may be based upon quite different profiles and sequence features. An alternative methodological approach would be to use bipartite networks [29, 30]. Unlike a typical ‘monopartite’ network which only encodes the degree of overall (dis)similarity among viruses, a bipartite network does not summarise virus similarity into a single number, but retains information about which genes viruses possess, and thus allows shared genes and/or horizontal gene transfers to be readily identified. These multi-dimensional relationships provide a fuller account of both genetic relatedness and the existence of modularity of different gene blocks in virus evolution [30, 31].

Pragmatically, however, GRAViTy was able to reproduce taxonomy relationships quite effectively despite potential problems of mosaic genomes (Table 1). This ‘monopartite’ approach was similarly successful in analysing sequence relationships among prokaryotic viruses using a clustering algorithm based on shared gene profiles [32]. In this latter study, viral clusters showed a reasonable match to their genus assignments (75% concordance). As the authors discussed, this comparative exercise was, however, hampered by the incomplete nature of phage taxonomy, the under-sampling of many existing taxa and a potentially greater degree of gene exchange in many groups of phage that distorts simpler metrics of virus relatedness. A methodological comparison with GRAViTy would be of considerable value in evaluating the effectiveness of these two related methodological approaches.

### Virus evolution and taxonomy

As in other areas of biology, virus classification strives to reproduce natural divisions that match evolutionary histories and degrees of genetic relatedness. In pursuing this for virus taxonomy, there is the necessary caveat that different types of virus may not share a common evolutionary origin that is separate from their hosts. A complete taxonomy of viruses is therefore always going to be an assemblage of several groups that are unrelated or not detectably related to each other, quite different from the monophyletic domains of eubacteria, archaea and eukaryotes and their ultimate hypothesised last common universal ancestor [33–35]. In general, the base-level groups identified by GRAViTy corresponded closely to those established by other means. To take the example of dsDNA viruses, it assigned viruses into five groups between which sequence similarity could not be detected. These groups were large DNA viruses (including NCLDVs), adenoviruses, Sputnik viruses (*Lavidaviridae*), *Polydnaviridae* and a grouping of polyomaviruses and papillomaviruses, the latter linked through possession of homologous early proteins. In group II, relatedness through possession of a common *Rep* gene allowed most ssDNA circular viruses to be grouped together, leaving only parvoviruses and anelloviruses as separate groups, again consistent with virus relationships established by other means.

All RNA viruses and reverse transcribing viruses in Baltimore groups III–VII possess structurally homologous ‘right-handed’ group I viral RNA polymerases and reverse transcriptases and may therefore potentially share a common evolutionary origin for at least their replication module. However, this similarity extends to RNA and DNA polymerases of cellular origins [36], and it is not currently clear at what stage in the origins of viruses this diversification of polymerases occurred. The degree of similarity in viral RNA polymerases is indeed highly restricted and extends typically only to those amino acid sites associated with defined catalytic functions with little or no identifiable sequence similarity elsewhere, even for regions of the polymerase that are clearly homologous in protein secondary structure. While it is very clearly possible that all RNA viruses do share a common evolutionary origin, this was not detected by GRAViTy which divided RNA viruses into a large number of separate, apparently unrelated groups (eight in Baltimore group III, four in group IV, three in group V and one in group VI/VII). While the use of PPHMMs to detect protein homologies is widely regarded as an effective and highly sensitive method [37–39], our use of *E* values < 0.001 and percentage identities of > 30% were relatively conservative and designed to maximise specificity in its detection of distant protein homology [37, 40–42]. The use of lower thresholds may detect more distantly related genes but at the risk of introducing false homologies that would severely distort predicted virus taxonomic relationships.

The detected groupings did, however, correspond to, or incorporate, many of the order assignments in the current ICTV taxonomy, including *Tymo*-, *Nido*- and *Bunyavirales* and provide a tentative basis for further groupings of more closely related viruses, such as alpha-like viruses (Fig. 2). Instances where the classifier was unable to reproduce existing order assignments included the *Herpesvirales* and *Picornavirales*. In the former, their current order assignments are based on their common morphology, with a characteristic appearance of capsid and tegument proteins [12]. Their implied evolutionary relatedness was, however, not apparent on genomic analysis, with little or no detectable structural or non-structural protein sequence homology or commonality in genome organisational features. Similarly, their polymerase gene phylogeny was paraphyletic (Additional file 11: Figure S15; Additional file 13). Membership of the *Picornavirales* presents an analogous situation, being based upon a particular capsid morphology (pseudo *T* = 3; [11]) even though the phylogeny of their RdRp and other replicative proteins genes is interspersed with other positive-stranded viruses (such as *Caliciviridae* which possess structurally distinct virions). In both examples, relatedness in the classifier largely followed phylogeny relationships of genes for replicative proteins.

These observations bring in a wider question of whether the continued use of structure/morphology based classifications is compatible with the planned classification of viruses in metagenomic datasets that may lack information on these phenotypic attributes. At least in the medium term, virion structure-based taxonomy assignments may become increasingly impractical as knowledge of viral diversity expands through metagenomics approaches, irrespective of the great power of structure-based classification to discern evolutionary relationships that lie far beyond the resolution of current genomic sequence-based methods [43–47]. We can predict a similar impasse with the classification of many groups of bacteriophages that are currently primarily morphology-based without the genomics underpinning that would enable metagenomically derived bacteriophages to be added to their taxonomy.

That is not to say that this will always be a restriction. Ongoing improvements in predictive structure-based sequence alignment method and their potentially greater ability to detect homologies in protein structure for genes that possess no detectable primary amino acid sequence similarity may enable these much more distance evolutionary connections at the virion level to be detected [36, 47]. The power of multi-parameter classifications described in the current study and elsewhere [32] is that outputs from improved structure modelling methods can be directly slotted into the processing pipeline and virus relationships re-evaluated within the same overall computational framework. Such methods may greatly expand the depth of phylogenetic trees constructed from CGJ distances and reveal considerably more about their deeper evolutionary relationships, perhaps including those currently apparent by morphology alone [36, 43, 44, 47, 48].

### Reconstruction of the ICTV taxonomy at family level

GRAViTy proved remarkably effective at reproducing the current family assignments of eukaryotic viruses using genomic data alone. By using reference and training sets as controls to model classifier performance with real-world sequence data, the classification pipeline showed high sensitivity and accuracy for the assignment of known viruses to existing families and equally a reliable ability to not assign viruses that were not in the reference set (specificity) (Table 1). Of the handful of discrepancies between current assignments and groupings predicted by GRAViTy, most cases represented taxonomies that were clearly at variance with genetic relationships and often represent historical non-genomically based assignments. Many of these may indeed be subject to formal revision by the ICTV in the future. These included the divergent groupings of rubella virus (genus *Rubivirus*) from members of the rest of the *Togaviridae* family. Similarly, a potential reassignment of *Diadromus pulchellus* ascovirus 4a (DpAV-4a) to the *Iridoviridae* is likely [49] and supported by GRAViTy. Another major discrepancy was the polyphyletic nature of members of the *Reoviridae*, which fell into four separate groups even though classified into a single family in the ICTV taxonomy [50]. In this case, the phylogeny of the RdRp gene was similarly polyphyletic with members of the family interspersed with those of other dsRNA families. Furthermore, the two sub-families of *Reoviridae* differ substantially in both genome organisation and in virion morphology, features that would typically lead to the assignments of other dsRNA viruses to separate virus families. Although GRAViTy is only one of many currently available guides to reovirus classification, its depiction of the substantial diversity of members of this currently assigned family may be of value in a re-evaluation of its ICTV status and comparability with family divisions elsewhere among dsRNA viruses.

### Identification of potential new virus families

Having developed and validated the methodology underlying GRAViTy, we conducted a preliminary analysis of several of the recently published virus metagenomic datasets to investigate its ability to depict relationships at the family and higher levels relative to those of existing taxa. GRAViTy is just one of many tools that may be used in virus classification, and we do not propose any specific taxonomic assignments based on the current analysis; these should be corroborated by methods such as core gene phylogenies and more detailed analyses of assignments of their most closely related viruses by expert groups. However, the results obtained indicate that the method is readily capable of distinguishing between those viruses that might be assigned as further variants within known families from those that cluster separately and are formally assigned as new by GRAViTy (Table 2; Additional file 7: Table S7).

We have therefore generated a list of potential novel virus groups that might be taken further for investigation and potentially for formal classification by experts in their respective areas of viral taxonomy (Additional file 7: Table S7). Sub-groupings of metagenomic-derived viruses within existing families may similarly provide the basis for future genus assignments if corroborated by the established criteria used within these families.

## Conclusions

The current analysis and taxonomic identifications incorporated sequence information from all of the eukaryotic viruses present in the current ICTV taxonomy. Analysis of larger datasets of metagenomic-derived sequences creates further PPHMMs that augment the original dataset, and their step-wise incorporation as GRAViTy is used to analyse these wider datasets will therefore increase its powers of identification and taxon prediction. Increasing the dataset size will provide opportunities for re-training as these newer groups become formally incorporated into the ICTV taxonomy.

As described above, eukaryotic viruses represent the best explored and categorised set of viruses for development of programs such as GRAViTy, but its current abilities are circumscribed to these viruses—it is currently blind to the much larger diversity of bacterial and archaeal viruses that populate marine and other environments. Future comparative evaluation of other approaches to systematic phage and archaeal virus classification, such as vConTACT [32] with bipartite methods and GRAViTy will be helpful in the future development of a combined, comprehensive classification tool that can take on the vast diversity of the virosphere.

## Methods

### Datasets

Viral genomes and associated information were compiled from (i) the ICTV 2016 Master Species List 31V1.1 (MSL; https://talk.ictvonline.org/files/master-species-lists/); (ii) Virus Metadata Resource (https://talk.ictvonline.org/taxonomy/vmr/); (iii) newly assigned viruses from the ICTV Executive Committee meeting, Singapore, 2017; and (iv) the NCBI virus RefSeq database (https://www.ncbi.nlm.nih.gov/genomes/GenomesGroup.cgi?taxid=10239 January 2017). Our dataset comprised 3854 whole genomic records of viruses, sampled across 7 Baltimore groups, 12 orders, 103 families, and 472 genera. Taxonomic assignments followed those of the ICTV MSL and an extended list of viruses in the Virus Metadata Resource and Refseq databases.

### Protein profile hidden Markov model (PPHMM) databases

Six PPHMM databases were generated, one for each Baltimore classification group with group VI (RT-RNA viruses) and group VII (RT-DNA viruses) sharing the same database since their members show substantial protein similarities. For each database, we first extracted protein sequences from relevant GenBank records. If protein annotations were missing, we six-frame translated the genomes and split the translated sequences at every stop codon. We then searched for the first methionine (M) in each of the sequences to determine the start of hypothetical proteins. There were 141 GenBank files in total that lack protein annotations (I: 58 records, II: 21 records, III: 22 records, IV: 15 records, V: 24 records, VI and VII: 1 record).

We noted that many of these hypothetical proteins were likely artefacts and not actual virus proteins. Indeed, the majority of the predicted sequences were very short, < 10 amino acids long, necessitating a length filter to exclude these from downstream analyses. This filter was also applied to the annotated virus proteins to maintain the consistency and to minimise false-positive similarity detection. To determine an appropriate threshold value, sensitivity (retention of annotated genes) and specificity (exclusion of non-annotated genes) was compared for different minimum protein lengths in several example sequences. Measurable loss of annotated virus proteins (~ 1%) was observed when using a length threshold of 50 amino acids, but this also led to the inclusion of between 50 and 80% of non-annotated, likely artefactual, gene predictions in each viral genome sequence. For example, for the human betaherpesvirus 6A (Accession number: X83413), we would have retained all annotated genes at the 50 amino acid cut-off, but 67% of its predicted proteins, if the virus were not to be annotated, would have been a false positive. The more conservative cut-off at 100 amino acids, which was selected as our length threshold in the protein extraction step, led to 5–10% loss of annotated genes but increased specificity to > 75%.

After the protein sequence extraction, all-versus-all pairwise protein comparisons were then performed by using BLASTp 2.2.28 [51] with default parameters except for the alignment number, which was specified to 1,000,000 to ensure that all significant hits were retrieved (see https://www.ncbi.nlm.nih.gov/books/NBK279684/ for BLASTp default parameter values). Hits with *E* value > 0.001, percentage identity < 30% and query/subject coverage < 75% were discarded. These highly conservative cut-offs were used to ensure that the resultant protein alignments that underpin PPHMMs (see below) will be of high quality.

Protein clusters were subsequently determined based on BLASTp bit scores by using a Markov clustering (MCL) algorithm (MCL 12–135) [52] with default parameters. The MCL algorithm is an unsupervised cluster algorithm based on simulation of random walks through a graph [52]. Herein, a graph refers to a network of protein similarity, in which proteins are represented as nodes and their pairwise similarity are represented by edges weighted by BLASTp bit scores (see https://micans.org/mcl/ for more details and default parameter values). If multiple BLASTp hits were returned for a pair of proteins, only the bit score of the best hit was used in the clustering computation. Proteins within each cluster were then aligned by using MUSCLE [53], with the gap opening and gap extension cost of − 3.0 and − 0.0, respectively. Finally, PPHMMs were generated from the resultant alignments using *hmmbuild* function, implemented in HMMER 3.1b1 (http://hmmer.org/), with default settings.

### Genomic organisation model databases

Similar to PPHMM databases, six databases of GOMs were constructed, one for each Baltimore group. Each GOM represents a genomic organisation of a particular taxonomic group; it is a matrix with each row being a list (i.e. a vector) of the locations of protein-coding regions within a particular genome. To locate protein-coding regions within a genome, we six-frame translated the sequence, concatenated them and scanned it against the PPHMM database of its respective Baltimore group using *hmmscan* function, implemented in HMMER 3.1b1 (http://hmmer.org/) with ‘nobias’ option. Herein, locations of protein-coding regions were defined as the middle of the HMM hits, transformed so that they referred to the locations in the original (translated) sequence. Hits with conditional *E* values (c-Evalues) > 0.001 and/or with negative hidden Markov model (HMM) scores were discarded. If a PPHMM exhibited significant similarity to multiple regions within the genome, the middle of the best hit was used to define its location. Values of genes’ locations can be either positive or negative depending on which strand the genes were found on. Gene locations with positive values indicate that the coding regions can be read off directly from the input sequence, while negative values mean that the coding regions can be found on the complementary strand. If coding regions could not be detected in the sequence, their locations were set to zero.

### Feature annotators

Six annotators were built; each uses a PPHMM database and the corresponding GOM database as the main annotation machinery. They annotate the presence of protein-coding regions (PPHMM signature) and compute the degree of genomic organisation similarity between the virus of interest and various taxonomic groups available in the databases (GOM signature). To annotate a genome, the annotator six-frame translates the sequence, concatenates them to form a single sequence query and subsequently scans the query against the PPHMM database with *hmmscan* function, implemented in HMMER 3.1b1 (http://hmmer.org/) with ‘nobias’ option. Hits with c-Evalues > 0.001 and/or with negative scores were discarded. In the case of segmented genomes, annotators concatenated them, from the largest to smallest segments, to form a single genomic representation prior the annotation. For circular genomes, there was no need for us to determine a breakpoint to linearise them in this study as all genomes were directly obtained the NCBI database, and they were all already provided in a linear form.

The PPHMM score is used to quantify the presence of protein-coding regions, with zero meaning no significant similarity could be detected. If a PPHMM exhibits significant similarity to multiple regions, an overall score computed across the entire sequence by the program is used. A PPHMM signature of a particular virus is simply a list of similarity scores of its genes to various PPHMMs in the database at the amino acid level. Locations of the detected protein-coding regions (as defined above) are also recorded and used to construct the GOM signature. Each element of a GOM signature is the ‘distance correlation coefficient’ [54] between the locations of query’s protein-coding regions and a GOM. Locations of the genes that are both absent in the query and the GOM were ignored in the distance correlation computation. The value of distance correlation ranges between 0 and 1, and it is unaffected by the choice of the input strand.

### Pairwise (dis)similarity measurement between a pair of genomes

Herein, an overall similarity between two genomes is quantified by a geometric mean of generalised Jaccard similarity scores, computed for the PPHMM signature (*J*_p_), and the GOM signature (*J*_o_). We term this index ‘a composite generalised Jaccard (CGJ) similarity index’, $J=\sqrt{J_{\mathrm{p}}\times {J}_{\mathrm{o}}}$. A generalised Jaccard similarity index for the PPHMM signature *J*_p_, for example, can be computed as follows:

$$ {J}_{\mathrm{p}}\left(x,y\right)=\frac{\sum_i\min \left({x}_{\mathrm{i}},{y}_{\mathrm{i}}\right)}{\sum_{\mathrm{i}}\max \left({x}_{\mathrm{i}},{y}_{\mathrm{i}}\right)} $$

where *x* and *y* are two genomes, of which the possession of their protein-coding regions is represented by the PPHMM signatures (*x*_1_, *x*_2_, ⋯, *x*_*n*_) and (*y*_1_, *y*_2_, ⋯, *y*_*n*_), respectively. *J*_o_ is also defined in the same way. The value of *J* ranges between 0 and 1, and thus, the overall similarity *J* also ranges between 0 and 1. The degree of dissimilarity, i.e. the distance, between two genomes *x* and *y* is simply *D*(*x*, *y*) = 1 − *J*(*x*, *y*), also ranges between 0 and 1.

### UPGMA dendrogram construction

All dendrograms in this study were constructed from complete pairwise distance matrices, by using the UPGMA algorithm, implemented in *linkage* and *to_tree* methods available in *SciPy* python library (http://www.scipy.org/). Cophenetic correlation [55] was used to measure how well a dendrogram preserved the original pairwise distances. The calculation was performed using *cophenet* method, also available in *SciPy* python library.

### Dendrogram bootstrapping

The resultant dendrograms were bootstrapped to evaluate the robustness of their topology. To bootstrap a dendrogram, we first randomly sampled its underlining PPHMMs with replacement such that the size of the resampled PPHMM database was the same as the original one. Each PPHMM was sampled with equal probability. Virus genomes were then re-annotated, and the GOM database was reconstructed based on the resampled PPHMM database. Subsequently, a complete pairwise distance matric was recomputed in order to build a bootstrapped UPGMA dendrogram sample. The process was repeated 100 times to obtain a distribution of bootstrap dendrograms. We then computed bootstrap support for branches on the best-estimate UPGMA dendrogram by using SumTrees [56] based on the obtained dendrogram distribution.

It is possible that several low bootstrapped support values might be caused by empty profile sampling—i.e. all elements in the bootstrapped signature are zero, containing information pertaining only to genes that are absent from the genome. This can happen with small viruses, such as members of *Papilloma*- and *Polyomaviridae* in group I and *Geminiviridae* in group II, which may exhibit similarity to only a small number of PPHMMs.

To investigate this effect, we perform re-bootstrap analyses on several sub-clades in the dendrograms (see Figs. 2, 5, 6, 7, 8 and 9 for clades that were re-bootstrapped). In re-bootstrap analyses, ‘irrelevant’ PPHMMs—i.e. those that none of the viruses in the clade exhibit similarity to—was excluded from the analyses. GOMs of virus families outside the clade were also excluded from the analyses. The same protocol as described above was used in re-bootstrapping.

### Classifiers and taxonomic assignment evaluation

Six classifiers were built; each simply computes pairwise CGJ similarities between the virus of interest and all reference (training) viruses and classifies it to the taxonomic group of the reference virus to which it exhibits the highest similarity with (1-nearest neighbour algorithm).

To validate the candidate taxonomic assignment, we employed two-step evaluation protocol. In the first step, the evaluator checked whether or not the virus of interest is ‘similar enough’ to the proposed candidate group. The CGJ similarity threshold is group specific. To estimate the threshold for a particular taxonomic group, we built distributions of its inter-group and intra-group CGJ similarity scores (*n* ≤ 10,000) and computed the score that best separates the two distributions using the support vector machine (SVM) algorithm, with ‘balanced’ class weight option. The SVM algorithm used in this study was implemented in *SVC* function, available from *Scikit-learn* python library [57]. If the observed CGJ similarity is less than the threshold, the candidate taxonomic assignment is rejected, and the sample is immediately relabelled as ‘unclassified’; otherwise, the second step of the evaluation will be employed to further evaluate the candidate taxonomic assignment.

In the second step, an UPGMA dendrogram containing all reference viruses and the virus of interest is used, and the evaluator will look at its neighbourhood. The taxonomic proposal will be accepted if any of the following conditions are met:

i) The sister clade is composed entirely of the members of the proposed candidate taxonomic group
ii) The immediate outgroup is composed entirely of the members of the proposed candidate taxonomic group
iii) One of the two basal branches of its sister clade leading to a clade that is composed entirely of the members of the proposed candidate taxonomic group.

To best estimate the placement of viruses, if multiple viruses are to be analysed at the same time, a dendrogram containing all viruses of interest will be used. Furthermore, since there are six classifiers, there are possibilities that a virus might be assigned to multiple taxonomic groups by multiple sub-pipelines. In such cases, the finalised taxonomic assignment is the one associated with the highest CGJ similarity score.

### Feature importance

Mutual information (MI) is used to determine features that are predictive of current virus taxonomy. MI measures the mutual dependence between two variables, which, in this case, are virus taxonomic assignments and PPHMM scores. A MI score of 0 means that the two variables are independent; otherwise, it has a positive value. The greater the value, the higher the dependency. *mutual_info_classif* method, which is available in *Scikit-learn* python library [57], was used to estimate MI scores. This method is stochastic however, and thus, mean values computed from 100 estimates were used in the result interpretation. We also noted that the sample size per taxonomic class can affect the MI score calculation, and the number of viruses can vary greatly from groups to groups. To take this into account, we sampled at most only two viruses from each taxonomic group in each of the 100 instances of the MI calculation.

Features that are shared by viruses in a particular set of families were also determined through inspection of MI scores. In this case, viruses that belong to the families of interest were relabelled so that they were in the same group, and the rest were in another separate group. Features that were not associated with the investigated families were removed from the analyses. As described above, mean MI values computed from 100 MI estimates were used in the result interpretation, and in each of the 100 instances of the MI calculation, at most only two viruses were sampled from each family.

We also examined whether or not non-structural and structural genes differ in their virus taxonomy predictive power. Features associated with polymerase and replicase (non-structural genes: replication) are labelled separately from other non-structural genes (non-structural genes: others), including genes encoding for reductase, kinase, T antigen, protease and helicase, as well as genes with their names containing the word ‘NS’, ‘non(-)structural’, ‘replication’ and ‘transcription’. Similarly, features associated with genes coding for viral particle shell proteins, i.e. capsid and gag genes, as well as those with their names containing the word ‘coat’, ‘shell’, ‘core’ and ‘nucleocapsid’, are labelled separately from other structural genes, including glycoprotein, matrix, tegument and envelop genes, and those with their names containing the word ‘surface’, ‘membrane’ and ‘structural’ (but not ‘non(-)structural’) (structural genes: Capsid/Gag and structural genes: others, respectively) (see Additional file 5: Table S5 for the values of MI scores).

### Classification of viruses derived from metagenomic data

A total of 2029 virus genomic records were compiled from six studies, reporting the discovery of diverse and unclassified small DNA and RNA viruses in metagenomic data [23–28]. Of these, 93 were already present in our reference dataset and therefore were excluded from the analysis. Furthermore, we noted that many of the RNA virus sequences were not whole genomes. We thus applied a length filter to exclude sequences that were obviously partial. The length thresholds were group specific. A threshold for a particular group was set to be the length of the smallest viruses in that group present in our reference database. To apply the length filter, it required to know which virus group these sequences belong to, and this information was derived from the initial taxonomic assignments obtained from the original studies. The final dataset contains 1381 viruses in total, which can be found in Additional file 7: Table S7. The length threshold can be found in Additional file 8: Table S8.

To classify these viruses, we ran the dataset through the GRAViTy pipeline using the PPHMM and GOM databases constructed from the reference viruses (Additional file 1: Table S1) (see the ‘Classifiers and taxonomic assignment evaluation’ section for details)*.* We also ran the dataset through the GRAViTy pipeline updated with genes from these unclassified viruses. Genes from viruses that did not pass through the length filter were also included in the updated PPHMM and GOM databases to maximise the power of virus classification. To update the databases, again, it required to know which Baltimore group these unclassified viruses belong to, and this information was derived from the initial taxonomic assignments proposed in the original studies. Finally, we manually inspected the heat maps (Figs. 5, 6, 7, 8 and 9) and dendrograms (Additional file 12: Figures S16–S19) depicting relationship of these unclassified viruses together with reference viruses to finalise the taxonomic assignments. Additional file 7: Table S7 summarises the results.

### Code availability

We wish to emphasise that our primary goal is to propose, investigate and evaluate a sequence-based framework for virus classification, rather than creating a ready-to-be-used bioinformatic tool. Nevertheless, the code and python scripts used in this study are available from GitHub: PAiewsakun/GRAViTy.

## Additional files

Additional file 1: **Table S1.** Viruses used in this study and their associated information. Listing of classified virus sequences used in the analysis. (XLSX 381 kb)

Additional file 2: **Table S2.** Summary of the virus taxa analysed in this study. Listing of ICTV assigned taxa of the sequences analysed in the study. (DOCX 17 kb)

Additional file 3: **Table S3.** Summary of protein profile hidden Markov model databases. Complete list and description of PPHMMs assigned from viral genome sequences. (XLSX 1181 kb)

Additional file 4: **Table S4.** Protein profile hidden Markov models responsible for inter-family relationships. List of PPHMMs that link together different virus families. (DOCX 23 kb)

Additional file 5: **Table S5.** Mutual information scores. Informativeness of different PPHMMs in the assignment of sequences to ICTV taxa expressed as a mutual information score. (XLSX 538 kb)

Additional file 6: **Table S6.** Cross-validation analysis. Scoring table for cross-validation analysis used to test specificity and sensitivity of GRAViTy. (XLSX 586 kb)

Additional file 7: **Table S7.** Classification of viruses from metagenomic data. A full listing of metagenomic sequences analysed by GRAViTy and their provisional assignments. (XLSX 110 kb)

Additional file 8: **Table S8.** Length thresholds used to identify (near) complete virus sequences in metagenomic datasets. Length thresholds used to exclude partial RNA virus sequences from metagenomic datasets. (XLSX 11 kb)

Additional file 9: **Figures S1–S6.** Heat maps of CGJ distances of classified viruses in Baltimore groups I–V and VI/VII. Versions of the summary heat maps shown in Fig. 1 with annotations for families and orders. (ZIP 2051 kb)

Additional file 10: **Figures S7–S12.** Dendrograms of individual virus sequences of classified viruses in Baltimore groups I–V and VI/VII. Full dendrograms that correspond to the collapsed dendrograms shown in Fig. 2. (ZIP 425 kb)

Additional file 11: **Figures S13–S15.** Phylogenetic trees of classified virus groups. Analysis of phylogeny relationships of viruses whose classification by GRAViTy conflicts with their ICTV assignments. (DOCX 825 kb)

Additional file 12: **Figures S16–S19.** Dendrograms of individual virus sequences of classified and metagenomic viruses in Baltimore groups II, III, IVa and IVb. Full dendrograms that correspond to the collapsed dendrograms shown in Figs. 5, 6, 7, 8 and 9 (lower panels). (ZIP 417 kb)

Additional file 13: Analysis of GRAViTy groupings that conflict with ITCV family assignments. Analysis of sequences whose assignment by GRAViTy conflicts with their ICTV classification. (PDF 59 kb)

## Abbreviations

CGJ: Composite generalised Jaccard
GOM: Genome organisation model
GRAViTy: Genome relationships applied to virus taxonomy
HTS: High-throughput sequencing
ICTV: International Committee on Taxonomy of Viruses
MI: Mutual information
MSL: Master Species List
PPHMM: Protein profile hidden Markov model
RdRp: RNA-dependent RNA polymerase
UTU: Unassigned taxonomy unit
