# Supplementary material for: The genomic underpinnings of eukaryotic virus taxonomy: creating a sequence-based framework for family-level virus classification
Source: Microbiome. 2018 Feb 20;6:38. doi: 10.1186/s40168-018-0422-7 (PMC5819261; doi:10.1186/s40168-018-0422-7)
Supplement: Supplementary file 2 — Table S2. Summary of the virus taxa analysed in this study. Listing of ICTV assigned taxa of the sequences analysed in the study. (DOCX 17 kb) [file 40168_2018_422_MOESM2_ESM.docx]

**Table S2. Summary of the viruses used in this study.** The numbers of viruses associated with each taxonomic group are in parentheses.

| **Baltimore classification** | **Order** | **Taxonomic assignment used in this study** | **Host*** |
| --- | --- | --- | --- |
| Group I: dsDNA viruses (586) | Herpesvirales (78) | Alloherpesviridae (7) | ver |
|  |  | Herpesviridae (69) | ver |
|  |  | Malacoherpesviridae (2) | inver |
|  | Unassigned | Adenoviridae (71) | ver |
|  | Unassigned | Ascoviridae (6) | inver |
|  | Unassigned | Asfarviridae (1) | ver, inver |
|  | Unassigned | Baculoviridae (76) | inver |
|  | Unassigned | Hytrosaviridae (2) | inver |
|  | Unassigned | Iridoviridae (21) | ver, inver |
|  | Unassigned | Lavidaviridae (3) | prot |
|  | Unassigned | Marseilleviridae (4) | inver, prot |
|  | Unassigned | Mimiviridae (2) | prot |
|  | Unassigned | Nimaviridae (1) | inver |
|  | Unassigned | Nudiviridae (6) | inver |
|  | Unassigned | Papillomaviridae (163) | ver |
|  | Unassigned | Phycodnaviridae (8) | algae |
|  | Unassigned | Polydnaviridae (4) | inver |
|  | Unassigned | Polyomaviridae (96) | ver |
|  | Unassigned | Poxviridae (44) | ver, inver |
| Group II: ssDNA viruses (1111) | Unassigned | Anelloviridae (94) | ver |
|  | Unassigned | Bacilladnaviridae (9) | algae |
|  | Unassigned | Bidnaviridae (1) | inver |
|  | Unassigned | Circoviridae (70) | ver, inver |
|  | Unassigned | Geminiviridae (699) | plants |
|  | Unassigned | Genomoviridae (73) | ver, inver, plants, fungi |
|  | Unassigned | Nanoviridae (11) | plants |
|  | Unassigned | Parvoviridae (111) | ver, inver |
|  | Unassigned | Smacoviridae (43) | ver, inver |
| Group III: dsRNA viruses (221) | Unassigned | Amalgaviridae (3) | plants |
|  | Unassigned | Birnaviridae (8) | ver, inver |
|  | Unassigned | Botybirnaviridae (1) | fungi |
|  | Unassigned | Chrysoviridae (7) | fungi |
|  | Unassigned | Endornaviridae (28) | plants, fungi |
|  | Unassigned | Hypoviridae (4) | fungi |
|  | Unassigned | Megabirnaviridae (1) | fungi |
|  | Unassigned | Partitiviridae (67) | plants, fungi, prot |
|  | Unassigned | Picobirnaviridae (2) | ver |
|  | Unassigned | Quadriviridae (1) | fungi |
|  | Unassigned | Reoviridae-Aquareovirus (5) | ver |
|  | Unassigned | Reoviridae-Cardoreovirus (1) | inver |
|  | Unassigned | Reoviridae-Coltivirus (2) | ver, inver |
|  | Unassigned | Reoviridae-Cypovirus (5) | inver |
|  | Unassigned | Reoviridae-Dinovernavirus (2) | inver |
|  | Unassigned | Reoviridae-Fijivirus (6) | inver, plants |
|  | Unassigned | Reoviridae-Mycoreovirus (2) | fungi |
|  | Unassigned | Reoviridae-Orbivirus (22) | ver, inver |
|  | Unassigned | Reoviridae-Orthoreovirus (11) | ver |
|  | Unassigned | Reoviridae-Oryzavirus (1) | inver, plants |
|  | Unassigned | Reoviridae-Phytoreovirus (3) | inver, plants |
|  | Unassigned | Reoviridae-Rotavirus (8) | ver |
|  | Unassigned | Reoviridae-Seadornavirus (3) | ver, inver |
|  | Unassigned | Totiviridae (28) | fungi, prot |
| Group IV: (+)ssRNA viruses (1253) | Nidovirales (82) | Arteriviridae (14) | ver |
|  |  | Coronaviridae (59) | ver |
|  |  | Mesoniviridae (8) | inver |
|  |  | Roniviridae (1) | inver |
|  | Picornavirales (266) | Dicistroviridae (21) | inver |
|  |  | Iflaviridae (31) | ver, inver |
|  |  | Marnaviridae (1) | algae |
|  |  | Picornaviridae (138) | ver |
|  |  | Polycipiviridae (14) | inver |
|  |  | Secoviridae (61) | plants |
|  | Tymovirales (182) | Alphaflexiviridae (53) | plants, fungi |
|  |  | Betaflexiviridae (90) | plants |
|  |  | Deltaflexiviridae (2) | plants |
|  |  | Gammaflexiviridae (1) | fungi |
|  |  | Tymoviridae (36) | inver, plants |
|  | Unassigned | Alphatetraviridae (3) | inver |
|  | Unassigned | Astroviridae (36) | ver |
|  | Unassigned | Barnaviridae (1) | fungi |
|  | Unassigned | Benyviridae (4) | plants |
|  | Unassigned | Bromoviridae (37) | plants |
|  | Unassigned | Caliciviridae (31) | ver |
|  | Unassigned | Carmotetraviridae (1) | inver |
|  | Unassigned | Closteroviridae (42) | plants |
|  | Unassigned | Flaviviridae (139) | ver, inver |
|  | Unassigned | Hepeviridae (5) | ver |
|  | Unassigned | Luteoviridae (42) | plants |
|  | Unassigned | Narnaviridae (7) | fungi |
|  | Unassigned | Nodaviridae (16) | ver, inver |
|  | Unassigned | Permutotetraviridae (1) | inver |
|  | Unassigned | Potyviridae (183) | plants |
|  | Unassigned | Sobemoviridae (20) | plants |
|  | Unassigned | Solinviviridae (2) | inver |
|  | Unassigned | Togaviridae (29) | ver, inver |
|  | Unassigned | Togaviridae-Rubivirus (1) | ver |
|  | Unassigned | Tombusviridae (73) | plants |
|  | Unassigned | Virgaviridae (50) | plants |
| Group V:  (-)ssRNA viruses (541) | Articulavirales (17) | Amnoonviridae (1) | ver |
|  |  | Orthomyxoviridae (16) | ver, inver |
|  | Bunyavirales (198) | Arenaviridae (40) | ver |
|  |  | Cruliviridae (1) | inver |
|  |  | Fimoviridae (9) | plants |
|  |  | Hantaviridae (32) | ver |
|  |  | Mypoviridae (1) | inver |
|  |  | Nairoviridae (18) | ver, inver |
|  |  | Peribunyaviridae (36) | ver, inver |
|  |  | Peribunyaviridae-Tospovirus (15) | inver, plants |
|  |  | Phasmaviridae (8) | inver |
|  |  | Phenuiviridae (37) | ver, inver, plants |
|  |  | Wupedeviridae (1) | inver |
|  | Goujianvirales (2) | Yueviridae (2) | inver |
|  | Jingchuvirales (30) | Chuviridae (30) | inver |
|  | Mononegavirales (281) | Artoviridae (7) | inver |
|  |  | Bornaviridae (13) | ver |
|  |  | Filoviridae (9) | ver |
|  |  | Mymonaviridae (6) | inver |
|  |  | Nyamiviridae (6) | ver, inver |
|  |  | Paramyxoviridae (60) | ver |
|  |  | Pneumoviridae (7) | ver |
|  |  | Rhabdoviridae (167) | ver, inver, plants |
|  |  | Sunviridae (1) | ver |
|  |  | Xinmoviridae (5) | inver |
|  | Muvirales (8) | Qinviridae (8) | inver |
|  | Serpentovirales (4) | Ophioviridae (4) | plants |
|  | Unassigned | Deltavirus (1) | ver |
| Group VI and VII: RT viruses (142) | Ortervirales (122) | Caulimoviridae (68) | plants |
|  |  | Retroviridae-Alpharetrovirus (3) | ver |
|  |  | Retroviridae-Betaretrovirus (9) | ver |
|  |  | Retroviridae-Deltaretrovirus (7) | ver |
|  |  | Retroviridae-Epsilonretrovirus (5) | ver |
|  |  | Retroviridae-Gammaretrovirus (13) | ver |
|  |  | Retroviridae-Lentivirus (11) | ver |
|  |  | Retroviridae-Spumavirus (6) | ver |
|  | Unassigned | Hepadnaviridae (20) | ver |

* ‘Ver’ = vertebrates; ‘Inver’ = invertebrates; ‘prot’ = protozoa
