# Supplementary material for: The genomic underpinnings of eukaryotic virus taxonomy: creating a sequence-based framework for family-level virus classification
Source: Microbiome. 2018 Feb 20;6:38. doi: 10.1186/s40168-018-0422-7 (PMC5819261; doi:10.1186/s40168-018-0422-7)
Supplement: Supplementary file 4 — Table S4. Protein profile hidden Markov models responsible for inter-family relationships. List of PPHMMs that link together different virus families. (DOCX 23 kb) [file 40168_2018_422_MOESM4_ESM.docx]

**Table S4 Protein profile Hidden Markov models responsible for inter-family relationships.**

| **Baltimore group** | **Taxonomic group** | **Feature (MI score)** |
| --- | --- | --- |
| Group I: dsDNA viruses | *Herpes-, Alloherpes-,* and *Malaco-herpesviridae (Herpesvirales)* | DNA packaging terminase subunit 1\|Herpesviridae (0.423),  DNA packaging terminase subunit 1\|Alloherpesviridae (0.345),  putative DNA packaging terminase\|Malacoherpesviridae (0.152) |
|  | *Papilloma-* and *Polyomaviridae* | large T antigen\|Polyomaviridae (0.201),  E1\|Papillomaviridae (0.189),  large T antigen\|Polyomaviridae (0.174),  E1\|Papillomaviridae (0.085) |
|  | *Herpes-, Alloherpes-, Malacoherpes-, Baculo-, Nudi-, Hytrosa-, Asco-, Irido-, Asfar-, Marseille-, Phycodna-, Pox-, Mimi-,* and *Nimaviridae* | EsV-1-93\|Phycodnaviridae (0.515),  DNA polymerase\|I_dsDNAviruses (0.506),  DNA polymerase catalytic subunit\|Herpesviridae (0.404),  DNA polymerase-like protein\|Hytrosaviridae (0.356),  DNA polymerase\|Poxviridae (0.301),  DNA polymerase\|Ascoviridae (0.270),  DNA polymerase\|Baculoviridae (0.268),  ribonucleotide reductase small subunit\|I_dsDNAviruses (0.267),  ribonucleotide reductase subunit 1\|Herpesviridae (0.260),  ribonucleotide reductase subunit 2\|Herpesviridae (0.256),  delta DNA polymerase (B family)\|Iridoviridae (0.249),  putative ribonucleotide reductase large subunit precursor\|Mimiviridae (0.248),  hypothetical protein\|Marseilleviridae (0.248),  ribonucleotide reductase large subunit\|I_dsDNAviruses (0.248),  DNA polymerase\|Mimiviridae (0.240),  ribonucleoside-diphosphate reductase large chain\|I_dsDNAviruses (0.238),  ribonucleotide reductase alpha subunit\|I_dsDNAviruses (0.232),  ribonucleotide reductase 1\|I_dsDNAviruses (0.224),  ribonucleoside-diphosphate reductase large chain precursor\|Iridoviridae (0.220),  ORF20\|Malacoherpesviridae (0.211),  Ribonucleoside-diphosphate reductase, beta subunit\|I_dsDNAviruses (0.200),  U28\|Herpesviridae (0.190),  protein kinase 1\|I_dsDNAviruses (0.185),  DNA polymerase family B\|Marseilleviridae (0.183),  UL40 ribonucleotide reductase subunit 2\|Herpesviridae (0.174),  DNA polymerase\|Baculoviridae (0.163),  serine/threonine protein kinase US3\|Herpesviridae (0.162),  Protein kinase\|Phycodnaviridae (0.161),  DNA polymerase\|Poxviridae (0.155) |
|  | *Polydnaviridae* (Bracovirus and Ichnovirus) | Hypothetical protein\|Polydnaviridae (0.152),  cysteine-rich protein\|Polydnaviridae (0.089),  CRP1 18.4\|Polydnaviridae (0.084),  cysteine motif gene-c4.1\|Polydnaviridae (0.083),  cysteine motif gene-d9.2\|Polydnaviridae (0.083),  cysteine motif gene-c19.1\|Polydnaviridae (0.083),  Hypothetical protein\|Polydnaviridae (0.070),  Hypothetical protein\|Polydnaviridae (0.070),  cysteine motif gene-d9.1\|Polydnaviridae (0.069),  VHv1.4 protein\|Polydnaviridae (0.068),  cysteine motif gene-d9.3\|Polydnaviridae (0.065) |
| Group II: ssDNA viruses | *Gemini-, Genomo-, Circo-, Smaco-, Nano-,* and *Bailladnaviridae* | master replication initiator protein\|Nanoviridae (0.164),  C2 protein\|II_ssDNAviruses (0.135),  RepA\|Geminiviridae (0.107),  replication associated protein\|Smacoviridae (0.098),  replication-associated protein\|Genomoviridae (0.096),  replication-associated protein\|Geminiviridae (0.094),  replication associated protein\|Smacoviridae (0.093),  putative replication-associated protein 1\|Circoviridae (0.092) |
| Group III: dsRNA viruses | *Chryso-, Quadri-, Megabirna-, Botybirna-* and *Totiviridae* | ssRNA-binding protein\|Totiviridae (0.430),  RNA-dependent RNA polymerase\|Totiviridae (0.429),  RNA dependent RNA polymerase\|Totiviridae (0.370),  RNA-dependent RNA polymerase\|Totiviridae (0.347),  cap-pol fusion protein\|Botybirnaviridae (0.343),  cap-pol fusion protein\|Totiviridae (0.339),  RNA-dependent RNA polymerase\|Chrysoviridae (0.333),  polyprotein\|Totiviridae (0.292),  RNA-dependent RNA polymerase\|Totiviridae (0.276),  RNA-dependent RNA polymerase\|Megabirnaviridae (0.274),  RNA dependent RNA polymerase\|Quadriviridae (0.252),  RNA-dependent RNA polymerase\|Totiviridae (0.197) |
|  | *Picobirna-, Amalga-,* and *Partitiviridae* | RNA-dependent RNA polymerase\|Partitiviridae (0.168),  putative RNA-dependent RNA polymerase\|Partitiviridae (0.056) |
| Group IV: (+)ssRNA viruses | *Alphaflexi-, Betaflexi-, Gammaflexi-, Deltaflexi-,* and *Tymoviridae (Tymovirales)* | replicase\|Gammaflexiviridae (0.410),  replication-associated polyprotein\|Deltaflexiviridae (0.410),  putative 239.6 kDa polyprotein\|Betaflexiviridae (0.405),  replicase\|Alphaflexiviridae (0.383),  polyprotein\|Tymoviridae (0.347),  RNA replicase\|Betaflexiviridae (0.320),  replicase\|Betaflexiviridae (0.310),  replicase\|Betaflexiviridae (0.305),  replicase\|Betaflexiviridae (0.305) |
|  | *Arteri-, Roni-, Corona-,* and *Mesoniviridae (Nidovirales)* | pp1a/b\|Coronaviridae (0.322),  replicase polyprotein 1ab\|Coronaviridae (0.321),  replicase polyprotein 1a\|Coronaviridae (0.307),  polyprotein 1ab\|Coronaviridae (0.264),  replicase polyprotein 1ab\|Coronaviridae (0.254),  replicase polyprotein\|Coronaviridae (0.204),  replicase polyprotein 1b\|Coronaviridae (0.183),  pp1ab\|Coronaviridae (0.172),  ORF1ab replicase polyprotein pp1ab\|Roniviridae (0.158),  orf1ab polyprotein\|Coronaviridae (0.155),  ORF1ab polyprotein\|Coronaviridae (0.143),  pp1b polyprotein\|Mesoniviridae (0.109),  ORF1ab\|Mesoniviridae (0.105),  putative 1b protein\|Arteriviridae (0.072),  ORF1b polyprotein\|Arteriviridae (0.023) |
|  | *Seco-, Marna-, Picorna-, Polycipi-, Dicistro-,* and *Iflaviridae (Picornavirales)* | polyprotein\|Iflaviridae (0.428),  polyprotein\|Iflaviridae (0.413),  polyprotein\|Iflaviridae (0.411),  nonstructural polyprotein\|Dicistroviridae (0.407),  polyprotein\|Secoviridae (0.394),  polyprotein 1\|Secoviridae (0.394),  polyprotein\|Picornaviridae (0.393),  polyprotein\|Iflaviridae (0.390),  polyprotein\|Iflaviridae (0.389),  nonstructural polyprotein\|Dicistroviridae (0.377),  polyprotein\|Secoviridae (0.374),  polyprotein\|Picornaviridae (0.373),  Hypothetical protein\|Polycipiviridae (0.368),  polyprotein\|Iflaviridae (0.368),  polyprotein\|Picornaviridae (0.367),  polyprotein\|Secoviridae (0.365),  nonstructural protein\|Dicistroviridae (0.363),  nonstructural polyprotein\|Dicistroviridae (0.360),  polyprotein\|Picornaviridae (0.357),  polyprotein\|Picornaviridae (0.353),  polyprotein\|Secoviridae (0.351),  nonstructural protein precursor\|Dicistroviridae (0.346),  polyprotein\|Picornaviridae (0.342),  RNA-dependent RNA polymerase\|Polycipiviridae (0.342),  non-structural polyprotein\|Dicistroviridae (0.337),  polyprotein\|Picornaviridae (0.336),  polyprotein\|Secoviridae (0.334),  polyprotein\|Secoviridae (0.333),  polyprotein\|Secoviridae (0.332),  polyprotein\|Picornaviridae (0.330),  polyprotein\|Picornaviridae (0.329),  polyprotein\|Secoviridae (0.325),  polyprotein\|Picornaviridae (0.323),  polyprotein\|Secoviridae (0.320),  Hypothetical protein\|Dicistroviridae (0.319),  polyprotein\|Picornaviridae (0.307),  polyprotein\|Picornaviridae (0.302),  polyprotein\|Picornaviridae (0.296),  hypothetical protein 3\|Polycipiviridae (0.270),  polyprotein P2-P3\|Picornaviridae (0.262),  Hypothetical protein\|Polycipiviridae (0.242),  polyprotein\|Marnaviridae (0.232),  250 kDa polyprotein\|Secoviridae (0.210),  capsid protein precursor\|Dicistroviridae (0.182),  Hypothetical protein\|Polycipiviridae (0.169),  capsid protein precursor\|Dicistroviridae (0.167),  capsid precursor\|Dicistroviridae (0.133),  hypothetical protein 3\|Polycipiviridae (0.127),  structural polyprotein\|Dicistroviridae (0.087),  Hypothetical protein\|Polycipiviridae (0.067),  polyprotein P1\|Picornaviridae (0.055),  capsid protein precursor\|Dicistroviridae (0.051) |
|  | *Seco-, Marna-, Picorna-, Polycipi-, Dicistro-, Ifla-, Solinvi-, Calici-,* and *Potyviridae* | polyprotein\|Picornaviridae (0.572),  polyprotein\|Picornaviridae (0.551),  polyprotein\|Picornaviridae (0.522),  polyprotein\|Picornaviridae (0.520),  polyprotein\|Picornaviridae (0.515),  polyprotein\|Picornaviridae (0.507),  polyprotein\|Caliciviridae (0.497),  polyprotein\|Iflaviridae (0.490),  non-structural polyprotein\|Dicistroviridae (0.484),  nonstructural polyprotein\|Dicistroviridae (0.479),  nonstructural polyprotein\|Dicistroviridae (0.469),  polyprotein\|Picornaviridae (0.468),  polyprotein\|Picornaviridae (0.467),  polyprotein\|Picornaviridae (0.461),  polyprotein\|Picornaviridae (0.460),  polyprotein\|Secoviridae (0.446),  polyprotein\|Iflaviridae (0.440),  polyprotein\|Secoviridae (0.438),  polyprotein 1\|Secoviridae (0.434),  polyprotein\|Secoviridae (0.432),  polyprotein\|Iflaviridae (0.425),  polyprotein\|Picornaviridae (0.424),  polyprotein\|Secoviridae (0.424),  polyprotein\|Iflaviridae (0.423),  polyprotein\|Iflaviridae (0.418),  nonstructural polyprotein\|Dicistroviridae (0.415),  polyprotein\|Secoviridae (0.414),  polyprotein\|Secoviridae (0.407),  RNA-dependent RNA polymerase\|Polycipiviridae (0.387),  polyprotein P2-P3\|Picornaviridae (0.379),  Hypothetical protein\|Polycipiviridae (0.377),  polyprotein\|Secoviridae (0.372),  polyprotein\|Secoviridae (0.371),  polyprotein\|Picornaviridae (0.362),  polyprotein\|Secoviridae (0.360),  hypothetical protein 3\|Polycipiviridae (0.353),  nonstructural protein precursor\|Dicistroviridae (0.348),  polyprotein\|Caliciviridae (0.343),  nonstructural protein\|Dicistroviridae (0.336),  non-structural polyprotein\|Caliciviridae (0.332),  polyprotein\|Iflaviridae (0.296),  Hypothetical protein\|Polycipiviridae (0.290),  polyprotein\|Picornaviridae (0.280),  putative nonstructural polyprotein\|Solinviviridae (0.279),  orf1\|Caliciviridae (0.268),  polyprotein\|Caliciviridae (0.266),  Hypothetical protein\|Dicistroviridae (0.264),  polyprotein\|Solinviviridae (0.253),  250 kDa polyprotein\|Secoviridae (0.233),  polyprotein\|Caliciviridae (0.228),  Hypothetical protein\|Polycipiviridae (0.205),  polyprotein\|Marnaviridae (0.171),  capsid protein precursor\|Dicistroviridae (0.137),  capsid protein precursor\|Dicistroviridae (0.135),  unnamed protein product\|Potyviridae (0.122),  hypothetical protein 3\|Polycipiviridae (0.105),  polyprotein\|Caliciviridae (0.104),  polyprotein\|Potyviridae (0.097),  capsid precursor\|Dicistroviridae (0.092),  polyprotein\|Potyviridae (0.087),  polyprotein\|Potyviridae (0.073),  polyprotein\|Potyviridae (0.066),  structural polyprotein\|Dicistroviridae (0.063),  putative NIb protein; nuclear inclusion b\|Potyviridae (0.062),  polyprotein P1\|Picornaviridae (0.049),  Hypothetical protein\|Polycipiviridae (0.049),  polyprotein\|Potyviridae (0.045),  capsid protein precursor\|Dicistroviridae (0.044),  VP1\|Caliciviridae (0.038),  putative capsid protein\|Polycipiviridae (0.029),  capsid protein precursor\|Caliciviridae (0.028) |
|  | *Bromo-, Virga-, Clostero-,* and *Togaviridae* | RNA-dependent RNA polymerase\|Closteroviridae (0.353),  putative RNA-dependent RNA polymerase\|Virgaviridae (0.303),  unknown protein\|Virgaviridae (0.284),  ORF 1a/1b fusion polyprotein\|Closteroviridae (0.283),  polyprotein 1a\|Closteroviridae (0.283),  187 kDa polymerase\|Virgaviridae (0.280),  replication protein\|Virgaviridae (0.280),  polyprotein\|Closteroviridae (0.280),  methyltransferase (MT) and helicase (HEL) domains\|Closteroviridae (0.278),  polyprotein 1a\|Closteroviridae (0.278),  nonstructural polyprotein\|Togaviridae (0.277),  RNA-dependent RNA polymerase\|Bromoviridae (0.273),  methyltransferase/helicase protein\|Closteroviridae (0.273),  replicase\|Bromoviridae (0.273),  replicase\|Virgaviridae (0.272),  ORF 1a/1b fusion polyprotein\|Closteroviridae (0.271),  ORF1a\|Closteroviridae (0.269),  1a protein\|Bromoviridae (0.267),  polyprotein 1a\|Closteroviridae (0.266),  2a protein\|Bromoviridae (0.265),  polymerase\|Bromoviridae (0.264),  methyltransferase/helicase polyprotein\|Closteroviridae (0.263),  349-kDa viral polyprotein\|Closteroviridae (0.263),  viral replicase\|Closteroviridae (0.262),  polyprotein 1a\|Closteroviridae (0.256),  p2 protein\|Bromoviridae (0.256),  2a protein\|Bromoviridae (0.256),  136K protein\|Virgaviridae (0.255),  p2 protein\|Bromoviridae (0.253),  putative polymerase\|Bromoviridae (0.253),  ORF1b\|Closteroviridae (0.252),  putative polymerase p2\|Bromoviridae (0.252),  126 kDa replicase\|Virgaviridae (0.248),  p182\|Closteroviridae (0.236),  polyprotein 1a\|Closteroviridae (0.223),  polyprotein\|Closteroviridae (0.213),  methyl transferase/helicase\|Closteroviridae (0.210),  polyprotein 1a\|Closteroviridae (0.197),  non-structural protein\|Togaviridae (0.156) |
|  | *Alphaflexi-, Betaflexi-, Gammaflexi-, Deltaflexi-, Tymo-, Bromo-, Virga-, Clostero-, Toga-, Hepe-, Alphatetra-, Benyviridae,* and *Togaviridae-Rubivirus* | polyprotein\|Hepeviridae (0.446),  RNA-dependent RNA polymerase\|Closteroviridae (0.403),  replicase\|Betaflexiviridae (0.383),  RNA-directed RNA polymerase\|Hepeviridae (0.301),  putative RNA-dependent RNA polymerase\|Virgaviridae (0.288),  viral replicase\|Closteroviridae (0.275),  ORF 1a/1b fusion polyprotein\|Closteroviridae (0.266),  replicase\|Gammaflexiviridae (0.241),  187 kDa polymerase\|Virgaviridae (0.238),  replicase\|Betaflexiviridae (0.232),  RNA replicase\|Betaflexiviridae (0.228),  1a protein\|Bromoviridae (0.224),  replicase\|Alphaflexiviridae (0.222),  ORF1b\|Closteroviridae (0.220),  replication protein\|Virgaviridae (0.217),  replicase\|Virgaviridae (0.213),  replicase\|Betaflexiviridae (0.212),  2a protein\|Bromoviridae (0.212),  126 kDa replicase\|Virgaviridae (0.211),  replicase\|Bromoviridae (0.196),  polyprotein\|Tymoviridae (0.196),  polyprotein 1a\|Closteroviridae (0.195),  polyprotein 1a\|Closteroviridae (0.189),  nonstructural polyprotein\|Togaviridae (0.184),  putative polymerase\|Bromoviridae (0.183),  ORF 1a/1b fusion polyprotein\|Closteroviridae (0.181),  polyprotein\|Closteroviridae (0.180),  136K protein\|Virgaviridae (0.176),  polyprotein 1a\|Closteroviridae (0.174),  polyprotein 1a\|Closteroviridae (0.168),  p182\|Closteroviridae (0.165),  ORF1a\|Closteroviridae (0.164),  349-kDa viral polyprotein\|Closteroviridae (0.158),  replication-associated polyprotein\|Deltaflexiviridae (0.156),  putative 239.6 kDa polyprotein\|Betaflexiviridae (0.154),  polyprotein 1a\|Closteroviridae (0.151) |
| Group V: (–)ssRNA viruses | *Amnoon-,* and *Orthomyxoviridae (Articulavirales)* | polymerase PB1\|Orthomyxoviridae (0.149),  hypothetical protein\|Amnoonviridae (0.029) |
|  | *Pneumo-, Sun-, Filo-, Paramyxo-, Rhabdo-, Arto-, Borna-, Mymona-, Nyami-,* and *Xinmoviridae (Mononegavirales)* | RNA dependent RNA polymerase\|Pneumoviridae (0.642),  L protein\|Rhabdoviridae (0.628),  RNA-dependent RNA polymerase\|Rhabdoviridae (0.626),  polymerase\|Filoviridae (0.619),  polymerase\|Rhabdoviridae (0.607),  RNA polymerase\|Paramyxoviridae (0.603),  L\|Rhabdoviridae (0.594),  RNA-dependent RNA polymerase\|Rhabdoviridae (0.581),  Large\|Sunviridae (0.577),  RNA-dependent RNA polymerase\|Mymonaviridae (0.573),  polymerase\|Rhabdoviridae (0.570),  RNA-dependent RNA polymerase\|Rhabdoviridae (0.568),  RNA-dependent RNA polymerase\|Xinmoviridae (0.565),  RNA-dependent RNA polymerase\|Xinmoviridae (0.565),  RNA-dependent RNA polymerase\|Mymonaviridae (0.565),  RNA-dependent RNA polymerase\|Rhabdoviridae (0.563),  putative RNA dependent RNA polymerase\|Nyamiviridae (0.560),  RNA-dependent RNA polymerase\|Artoviridae (0.560),  RNA-dependent RNA polymerase\|Rhabdoviridae (0.551),  L\|Rhabdoviridae (0.542),  polymerase\|Rhabdoviridae (0.542),  L-polymerase\|Bornaviridae (0.535),  RNA-dependent RNA polymerase\|Mymonaviridae (0.530),  RNA polymerase\|Rhabdoviridae (0.498),  RNA-dependent RNA polymerase\|Mymonaviridae (0.489),  RNA-dependent RNA polymerase\|Nyamiviridae (0.469),  RNA-dependent RNA polymerase\|Nyamiviridae (0.468) |
|  | *Pneumo-, Sun-, Filo-, Paramyxo-, Rhabdo-, Arto-, Borna-, Mymona-, Nyami-, Xinmo-,* and *Chuviridae* | putative RNA dependent RNA polymerase\|Nyamiviridae (0.689),  RNA-dependent RNA polymerase\|Artoviridae (0.689),  RNA-dependent RNA polymerase\|Xinmoviridae (0.689),  RNA-dependent RNA polymerase\|Xinmoviridae (0.688),  RNA-dependent RNA polymerase\|Mymonaviridae (0.688),  RNA-dependent RNA polymerase\|Rhabdoviridae (0.686),  RNA-dependent RNA polymerase\|Mymonaviridae (0.685),  RNA-dependent RNA polymerase\|Rhabdoviridae (0.684),  RNA-dependent RNA polymerase\|Rhabdoviridae (0.684),  L\|Rhabdoviridae (0.681),  RNA-dependent RNA polymerase\|Rhabdoviridae (0.681),  polymerase\|Rhabdoviridae (0.673),  L\|Rhabdoviridae (0.672),  RNA polymerase\|Paramyxoviridae (0.666),  RNA-dependent RNA polymerase\|Rhabdoviridae (0.666),  RNA-dependent RNA polymerase\|Mymonaviridae (0.664),  polymerase\|Rhabdoviridae (0.657),  L protein\|Rhabdoviridae (0.648),  polymerase\|Rhabdoviridae (0.638),  L-polymerase\|Bornaviridae (0.631),  polymerase\|Chuviridae (0.626),  RNA polymerase\|Rhabdoviridae (0.625),  polymerase\|Filoviridae (0.615),  RNA-dependent RNA polymerase\|Nyamiviridae (0.601),  RNA-dependent RNA polymerase\|Mymonaviridae (0.601),  RNA-dependent RNA polymerase\|Nyamiviridae (0.596),  RNA dependent RNA polymerase\|Pneumoviridae (0.595),  Large\|Sunviridae (0.584),  RNA-dependent RNA polymerase\|Chuviridae (0.552),  RNA-dependent RNA polymerase\|Chuviridae (0.497),  RNA-dependent RNA polymerase\|Chuviridae (0.488),  polymerase\|Chuviridae (0.453),  RNA-dependent RNA polymerase\|Chuviridae (0.421),  RNA-dependent RNA polymerase\|Chuviridae (0.377),  polymerase\|Chuviridae (0.283),  polymerase\|Chuviridae (0.249),  RNA-dependent RNA polymrease\|Chuviridae (0.161) |
|  | *Nairo-, Wupede-, Arena-, Mypo-, Phenui-, Phasma-, Hanta-, Fimo-, Tospo-, Cruli-,* and *Peribunyaviridae* (*Bunyavirales*) | RNA-dependent RNA polymerase\|Phenuiviridae (0.306),  RNA-dependent RNA polymerase\|Phasmaviridae (0.227),  RNA-dependent RNA polymerase\|Peribunyaviridae (0.224),  RNA-dependent RNA polymerase\|Cruliviridae (0.211),  polymerase\|Phenuiviridae (0.209),  RNA-dependent RNA polymerase\|Hantaviridae (0.209),  L protein\|Tospoviridae (0.202),  RNA dependent RNA polymerase\|Fimoviridae (0.190),  RNA-dependent RNA polymerase\|Phenuiviridae (0.179),  RNA-dependent RNA polymerase\|Phasmaviridae (0.143),  RNA-dependent RNA polymerase\|Phasmaviridae (0.125),  L protein\|Arenaviridae (0.120),  RNA-dependent RNA polymerase\|Wupedeviridae (0.112),  RNA-dependent RNA polymerase\|Mypoviridae (0.108),  polyprotein\|Peribunyaviridae (0.102),  glycoprotein precursor\|Tospoviridae (0.098),  RNA-dependent RNA polymerase\|Phenuiviridae (0.097),  glycoprotein precursor\|Peribunyaviridae (0.097),  L protein\|Arenaviridae (0.092),  putative glycoprotein\|Cruliviridae (0.091),  Hypothetical protein\|Nairoviridae (0.069),  glycoprotein precursor\|Nairoviridae (0.063),  glycoprotein\|Hantaviridae (0.058),  RNA-dependent RNA polymerase\|Nairoviridae (0.054),  glycoprotein precursor\|Nairoviridae (0.052),  RNA-dependent RNA polymerase\|Nairoviridae (0.052),  RNA Polymerase\|Phenuiviridae (0.051),  glycoprotein precursor\|Peribunyaviridae (0.043),  glycoprotein precursor\|Nairoviridae (0.043),  glycoprotein precursor\|Phenuiviridae (0.042) |
| Group VI and VII: RT viruses | *Retroviridae* (Alpha-, Beta-, Delta-, Epsilon-, Gammaretrovirus, Lentivirus, Spumavirus) | pol polyprotein\|Retroviridae-Lentivirus (0.507),  pol polyprotein\|VI_VII_RTviruses (0.505),  pol protein\|VI_VII_RTviruses (0.488),  polymerase\|Retroviridae-Deltaretrovirus (0.487),  Pr180\|Retroviridae-Gammaretrovirus (0.483),  reverse transcriptase/envelope protein\|Retroviridae-Deltaretrovirus (0.473),  unnamed protein product; coding sequence of pol\|Retroviridae-Gammaretrovirus (0.463),  Pr180 polyprotein precursor\|VI_VII_RTviruses (0.430),  gag-pro-pol polyprotein\|Retroviridae-Epsilonretrovirus (0.417),  gag-pro-pol polyprotein\|Retroviridae-Deltaretrovirus (0.413),  Pr66\|Retroviridae-Deltaretrovirus (0.204),  gag polyprotein\|Retroviridae-Deltaretrovirus (0.202),  gag polyprotein\|Retroviridae-Lentivirus (0.155),  gag protein\|Retroviridae-Lentivirus (0.151),  gag protein\|Retroviridae-Betaretrovirus (0.145),  gag protein\|Retroviridae-Lentivirus (0.139),  pol\|Retroviridae-Spumavirus (0.136),  gag protein\|Retroviridae-Lentivirus (0.134),  envelope protein\|Retroviridae-Gammaretrovirus (0.130),  Pr110\|Retroviridae-Betaretrovirus (0.129),  envelope glycoprotein\|VI_VII_RTviruses (0.103),  putative group-specific antigen\|Retroviridae-Betaretrovirus (0.096),  envelope glycoprotein\|Retroviridae-Deltaretrovirus (0.086),  envelope protein\|Retroviridae-Alpharetrovirus (0.081),  gag polyprotein\|Retroviridae-Alpharetrovirus (0.072),  protease\|Retroviridae-Betaretrovirus (0.063),  gag protein\|Retroviridae-Gammaretrovirus (0.054) |
|  | *Retro-,* and *Caulimoviridae (Ortervirales)* | pol\|Retroviridae-Spumavirus (0.352),  gag-pro-pol polyprotein\|Retroviridae-Epsilonretrovirus (0.327),  Pr180\|Retroviridae-Gammaretrovirus (0.313),  pol polyprotein\|VI_VII_RTviruses (0.309),  ORF I polyprotein\|Caulimoviridae (0.279),  pol polyprotein\|Retroviridae-Lentivirus (0.274),  gag-pro-pol polyprotein\|Retroviridae-Deltaretrovirus (0.205),  polymerase\|Retroviridae-Deltaretrovirus (0.201),  Pr180 polyprotein precursor\|VI_VII_RTviruses (0.191),  unnamed protein product; coding sequence of pol\|Retroviridae-Gammaretrovirus (0.164),  pol protein\|VI_VII_RTviruses (0.162),  polyprotein\|Caulimoviridae (0.158),  replicase\|Caulimoviridae (0.153),  polyprotein\|Caulimoviridae (0.148) |
