# Supplementary figures and images for: The genomic underpinnings of eukaryotic virus taxonomy: creating a sequence-based framework for family-level virus classification
Source: Microbiome. 2018 Feb 20;6:38. doi: 10.1186/s40168-018-0422-7 (PMC5819261; doi:10.1186/s40168-018-0422-7)

Pairwise distance matrix Group I: dsDNA viruses

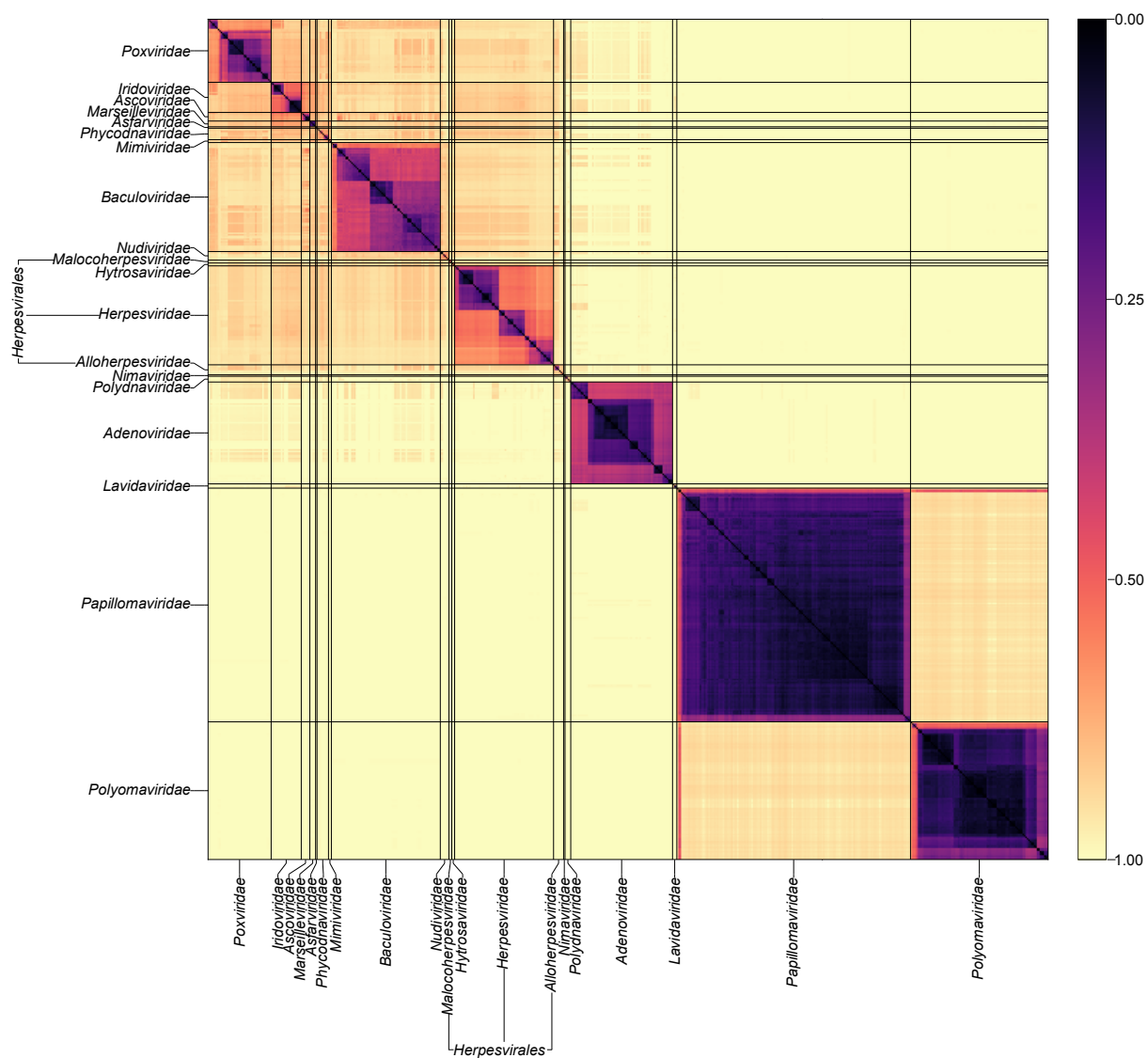

Supplement: Supplementary file 9 — Figures S1–S6. Heat maps of CGJ distances of classified viruses in Baltimore groups I–V and VI/VII. Versions of the summary heat maps shown in Fig. 1 with annotations for families and orders. (ZIP 2051 kb) [file 40168_2018_422_MOESM9_ESM.zip › Figure S1.pdf]

Pairwise distance matrix Group II: ssDNA viruses

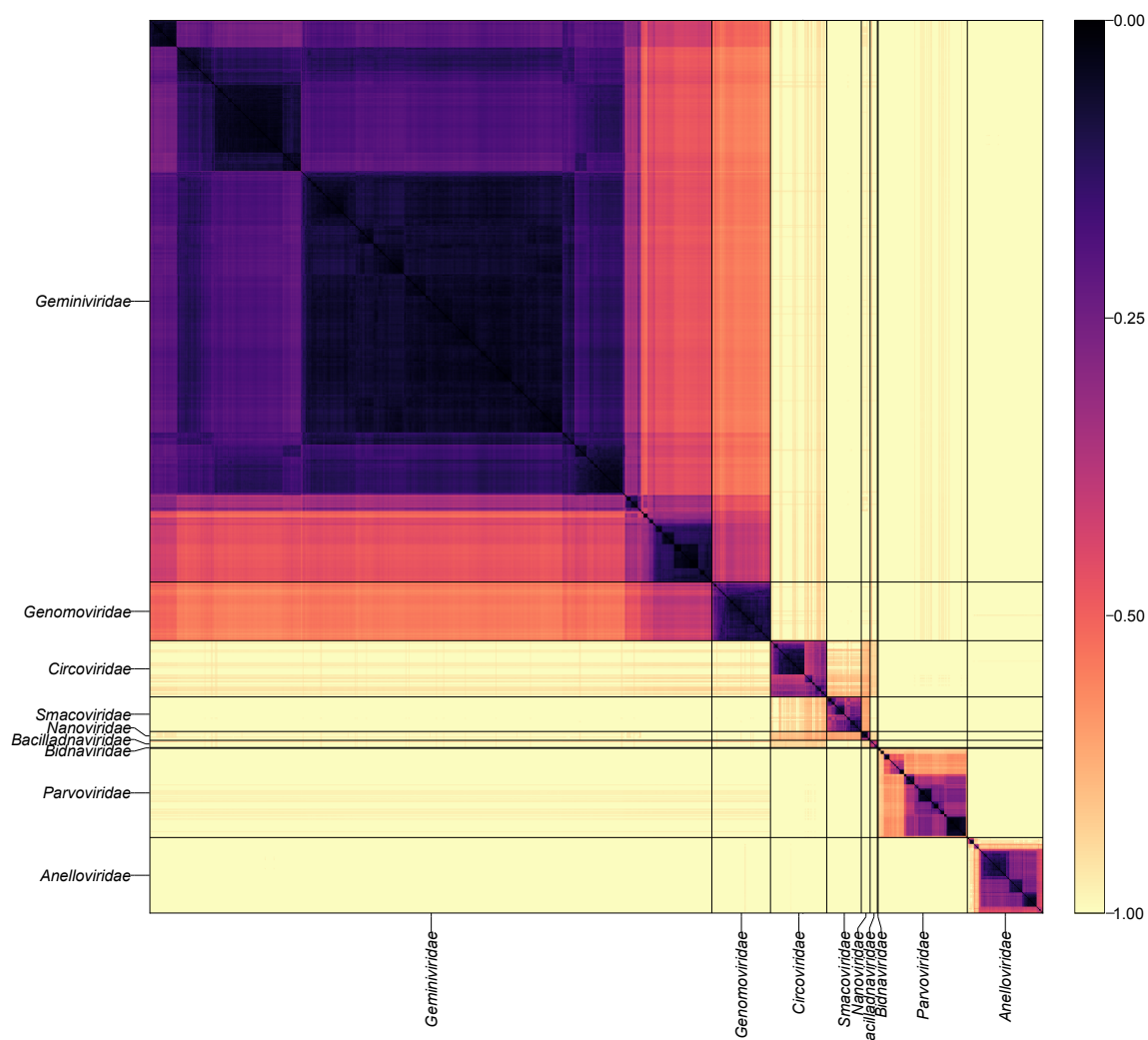

Supplement: Supplementary file 9 — Figures S1–S6. Heat maps of CGJ distances of classified viruses in Baltimore groups I–V and VI/VII. Versions of the summary heat maps shown in Fig. 1 with annotations for families and orders. (ZIP 2051 kb) [file 40168_2018_422_MOESM9_ESM.zip › Figure S2.pdf]

Pairwise distance matrix Group III: dsRNA viruses

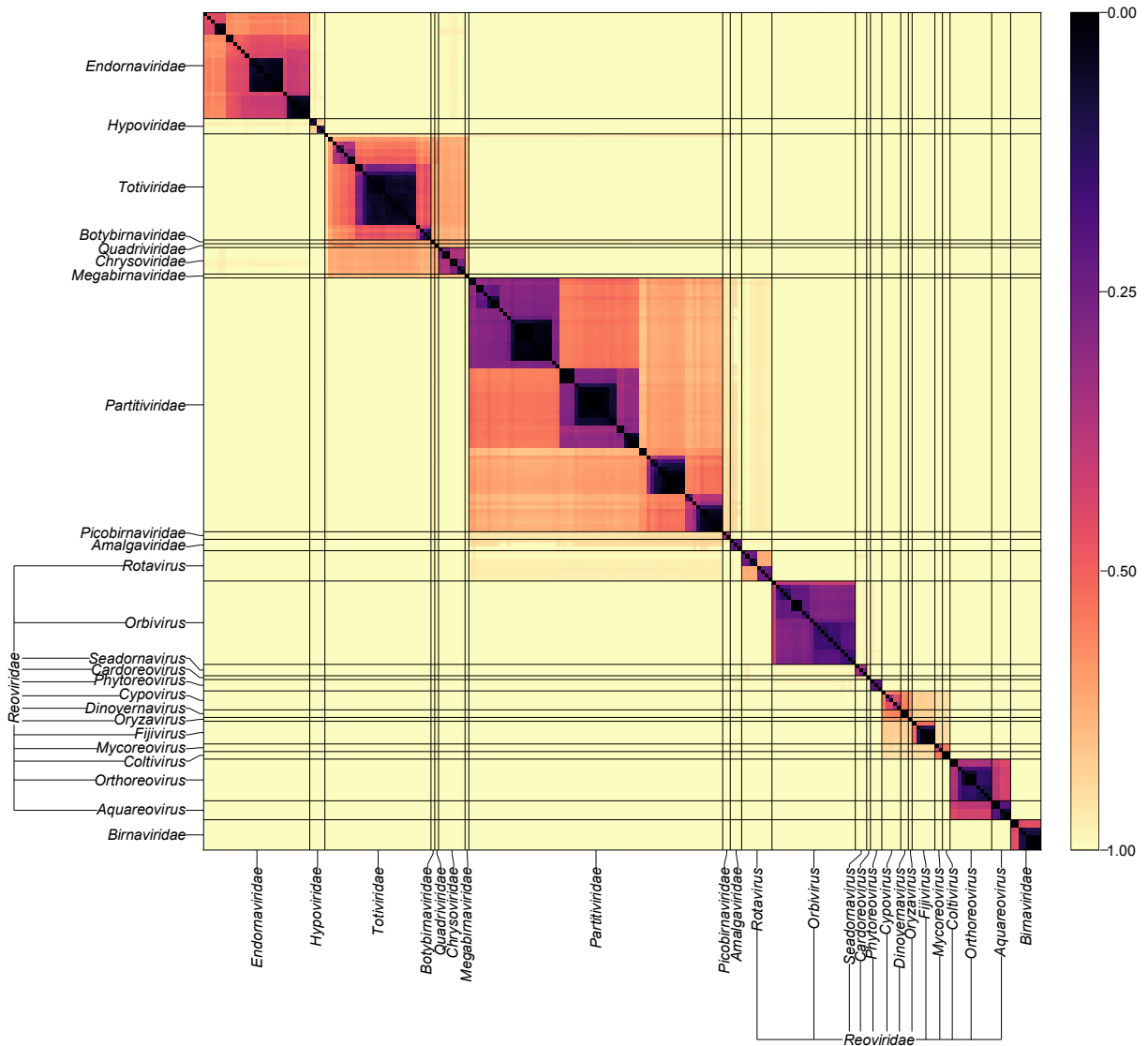

Supplement: Supplementary file 9 — Figures S1–S6. Heat maps of CGJ distances of classified viruses in Baltimore groups I–V and VI/VII. Versions of the summary heat maps shown in Fig. 1 with annotations for families and orders. (ZIP 2051 kb) [file 40168_2018_422_MOESM9_ESM.zip › Figure S3.pdf]

Pairwise distance matrix Group IV: (+)ssRNA viruses

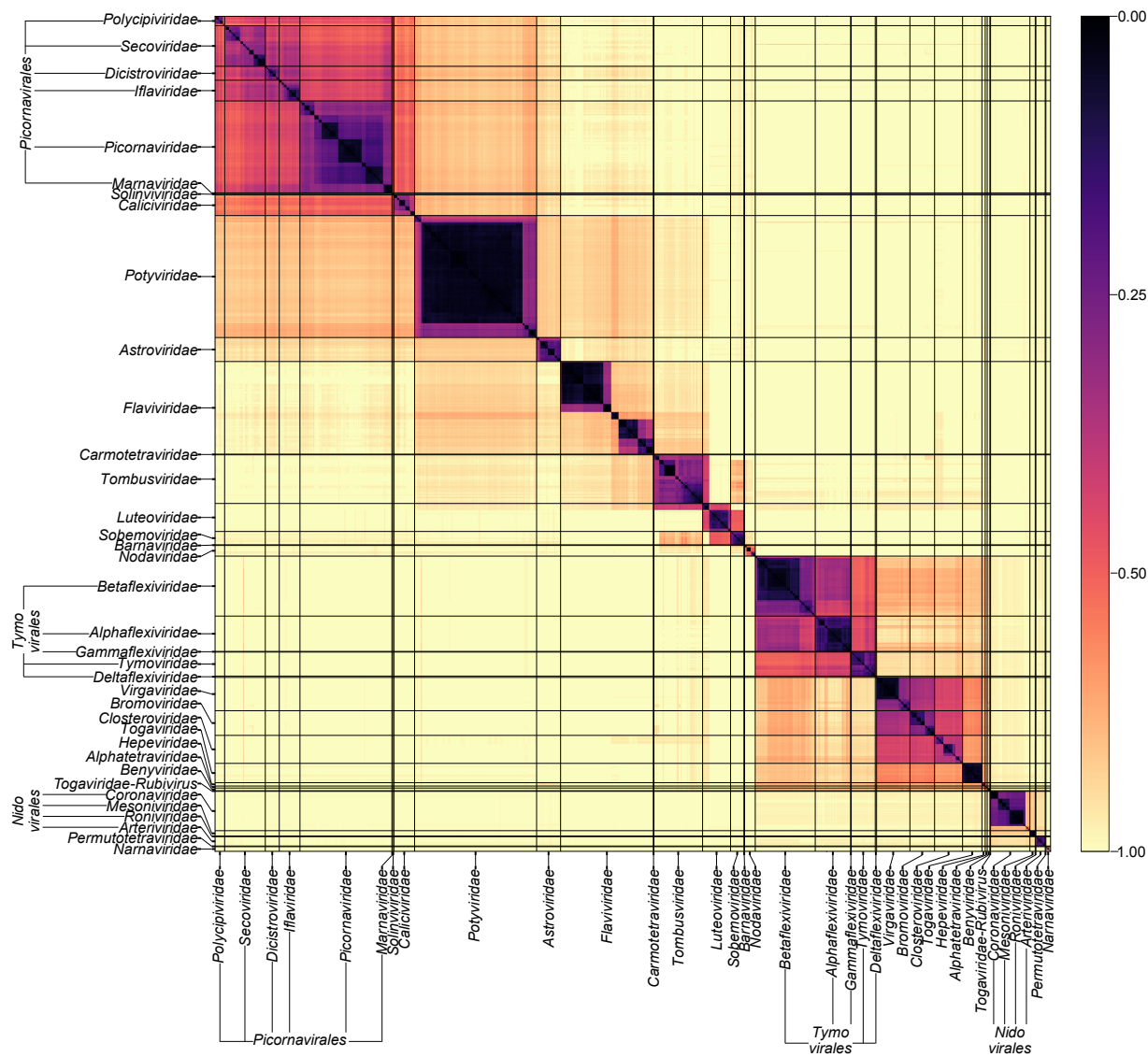

Supplement: Supplementary file 9 — Figures S1–S6. Heat maps of CGJ distances of classified viruses in Baltimore groups I–V and VI/VII. Versions of the summary heat maps shown in Fig. 1 with annotations for families and orders. (ZIP 2051 kb) [file 40168_2018_422_MOESM9_ESM.zip › Figure S4.pdf]

Pairwise distance matrix Group V: (-)ssRNA viruses

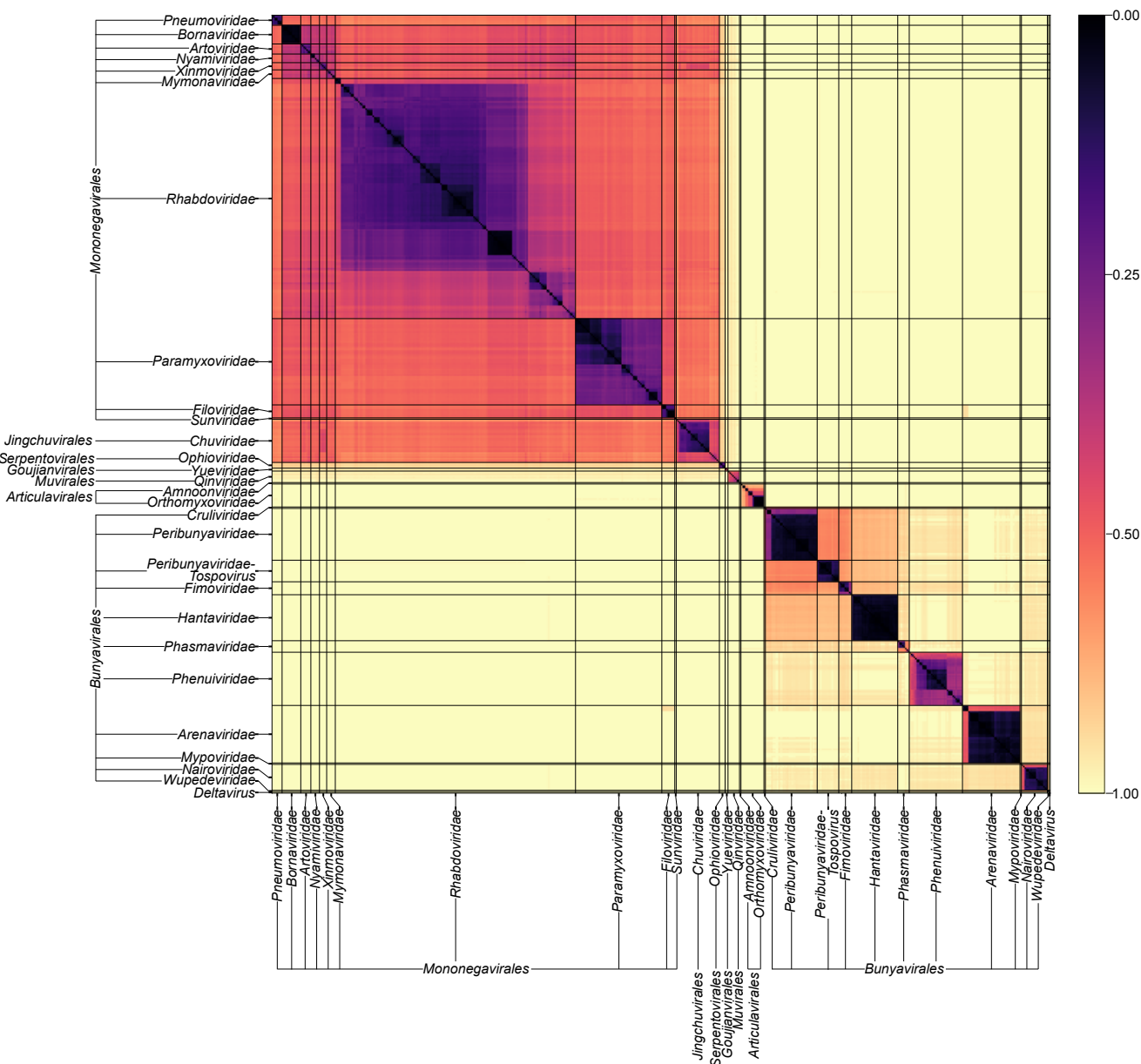

Supplement: Supplementary file 9 — Figures S1–S6. Heat maps of CGJ distances of classified viruses in Baltimore groups I–V and VI/VII. Versions of the summary heat maps shown in Fig. 1 with annotations for families and orders. (ZIP 2051 kb) [file 40168_2018_422_MOESM9_ESM.zip › Figure S5.pdf]

Pairwise distance matrix Group VI&VII: RT viruses

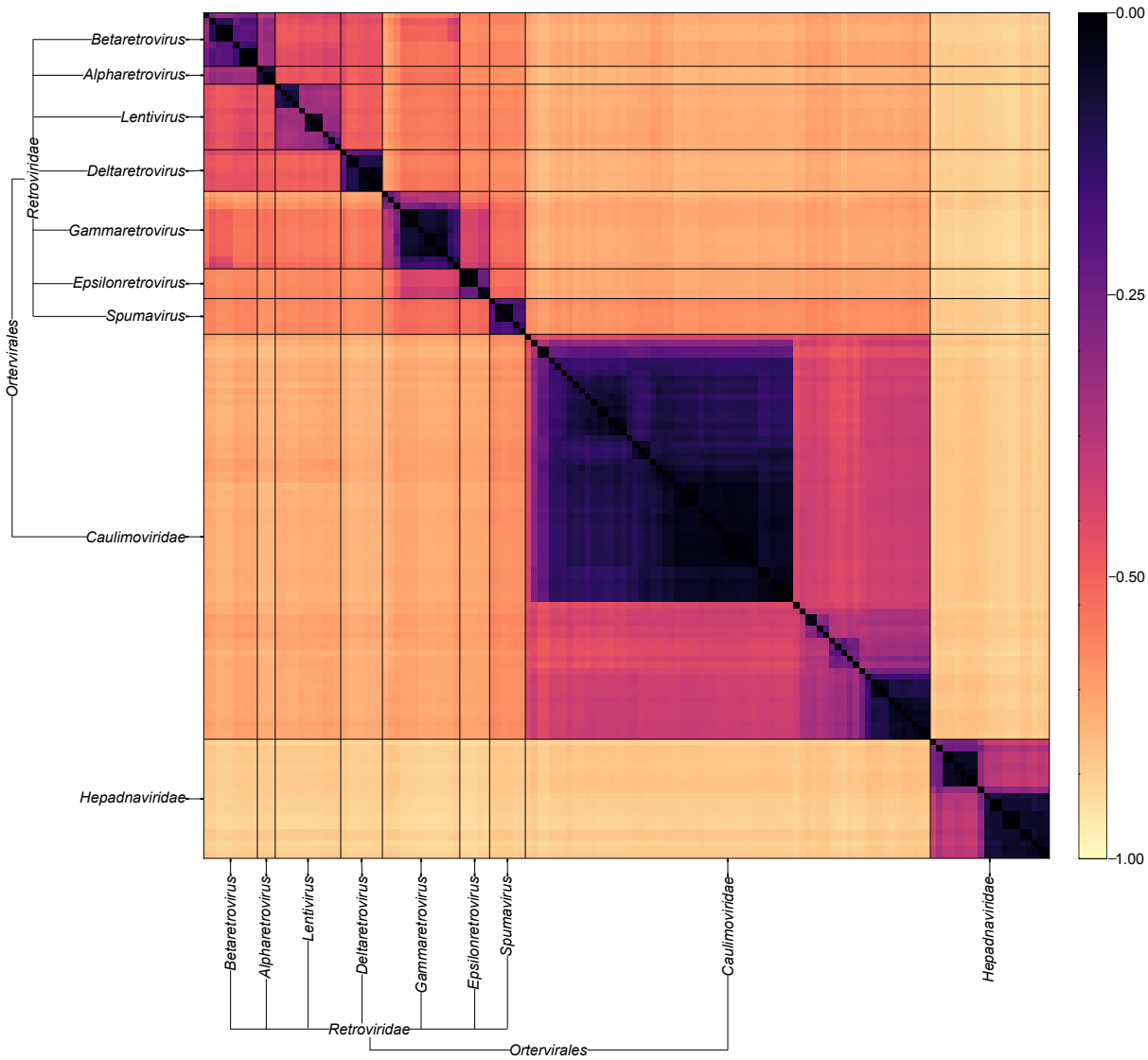

Supplement: Supplementary file 9 — Figures S1–S6. Heat maps of CGJ distances of classified viruses in Baltimore groups I–V and VI/VII. Versions of the summary heat maps shown in Fig. 1 with annotations for families and orders. (ZIP 2051 kb) [file 40168_2018_422_MOESM9_ESM.zip › Figure S6.pdf]

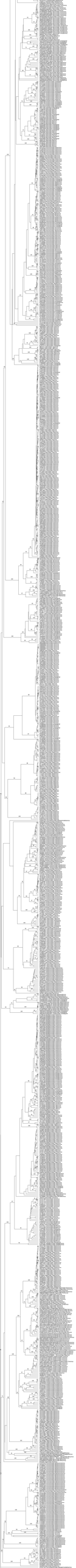

Supplement: Supplementary file 10 — Figures S7–S12. Dendrograms of individual virus sequences of classified viruses in Baltimore groups I–V and VI/VII. Full dendrograms that correspond to the collapsed dendrograms shown in Fig. 2. (ZIP 425 kb) [file 40168_2018_422_MOESM10_ESM.zip › Figure S10.pdf]

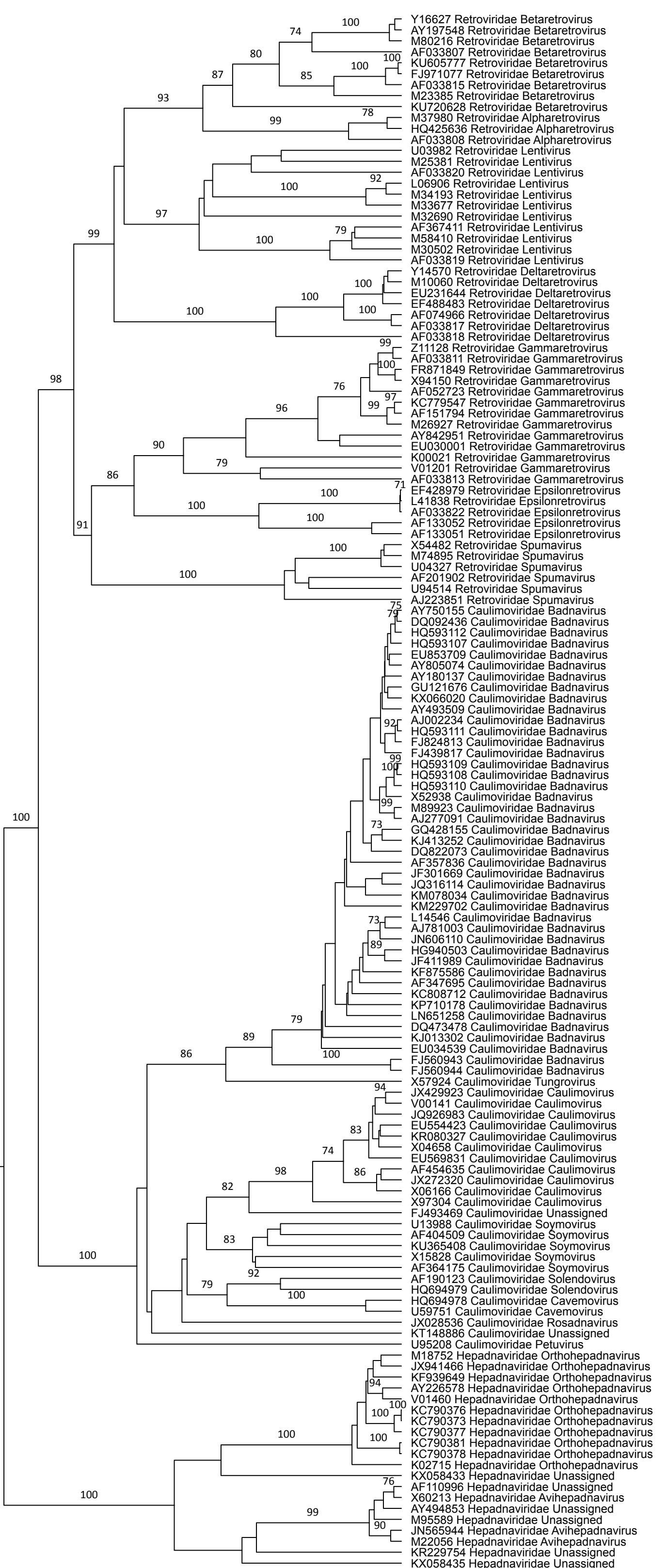

0.09

Supplement: Supplementary file 10 — Figures S7–S12. Dendrograms of individual virus sequences of classified viruses in Baltimore groups I–V and VI/VII. Full dendrograms that correspond to the collapsed dendrograms shown in Fig. 2. (ZIP 425 kb) [file 40168_2018_422_MOESM10_ESM.zip › Figure S12.pdf]

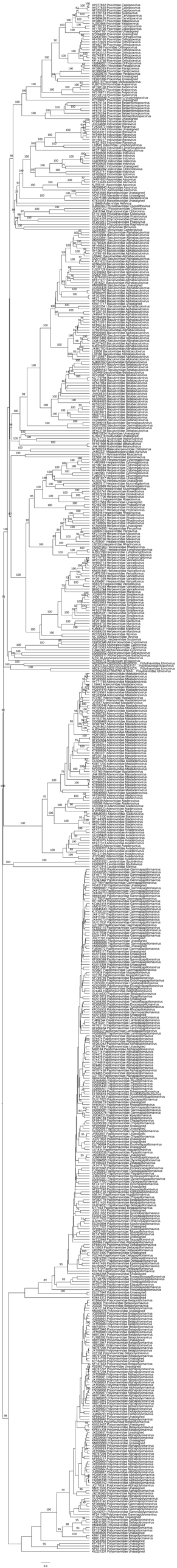

Supplement: Supplementary file 10 — Figures S7–S12. Dendrograms of individual virus sequences of classified viruses in Baltimore groups I–V and VI/VII. Full dendrograms that correspond to the collapsed dendrograms shown in Fig. 2. (ZIP 425 kb) [file 40168_2018_422_MOESM10_ESM.zip › Figure S7.pdf]

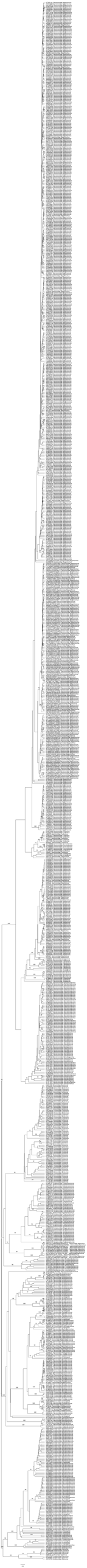

Supplement: Supplementary file 10 — Figures S7–S12. Dendrograms of individual virus sequences of classified viruses in Baltimore groups I–V and VI/VII. Full dendrograms that correspond to the collapsed dendrograms shown in Fig. 2. (ZIP 425 kb) [file 40168_2018_422_MOESM10_ESM.zip › Figure S8.pdf]

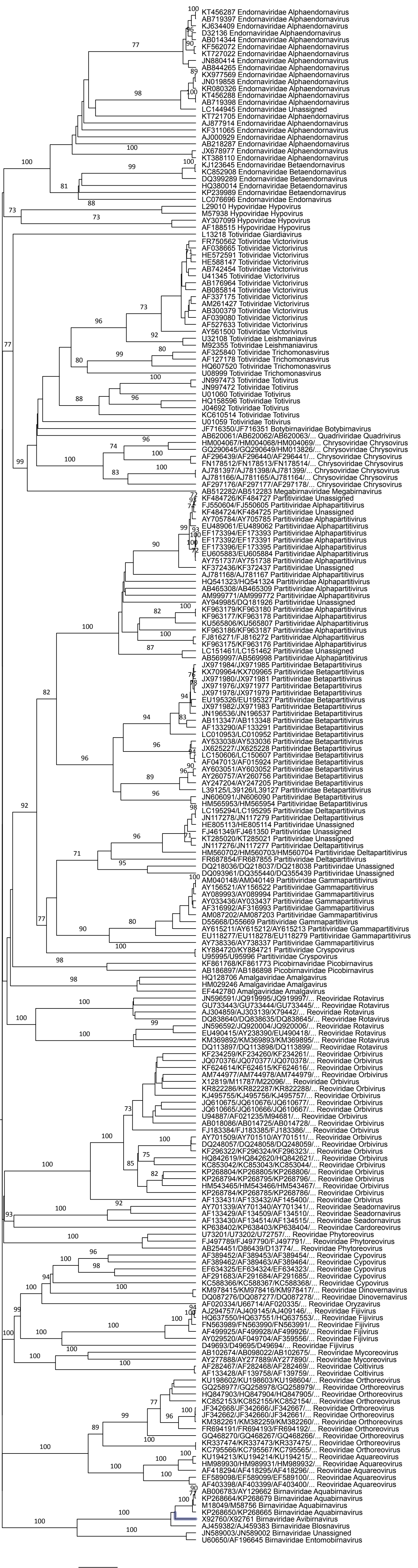

Supplement: Supplementary file 10 — Figures S7–S12. Dendrograms of individual virus sequences of classified viruses in Baltimore groups I–V and VI/VII. Full dendrograms that correspond to the collapsed dendrograms shown in Fig. 2. (ZIP 425 kb) [file 40168_2018_422_MOESM10_ESM.zip › Figure S9.pdf]

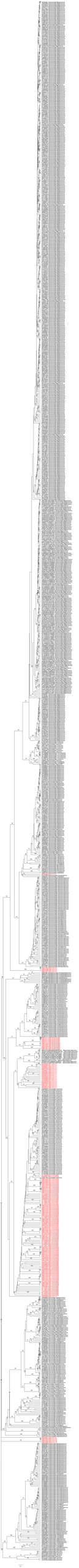

Supplement: Supplementary file 12 — Figures S16–S19. Dendrograms of individual virus sequences of classified and metagenomic viruses in Baltimore groups II, III, IVa and IVb. Full dendrograms that correspond to the collapsed dendrograms shown in Figs. 5, 6, 7, 8 and 9 (lower panels). (ZIP 417 kb) [file 40168_2018_422_MOESM12_ESM.zip › Figure S16.pdf]

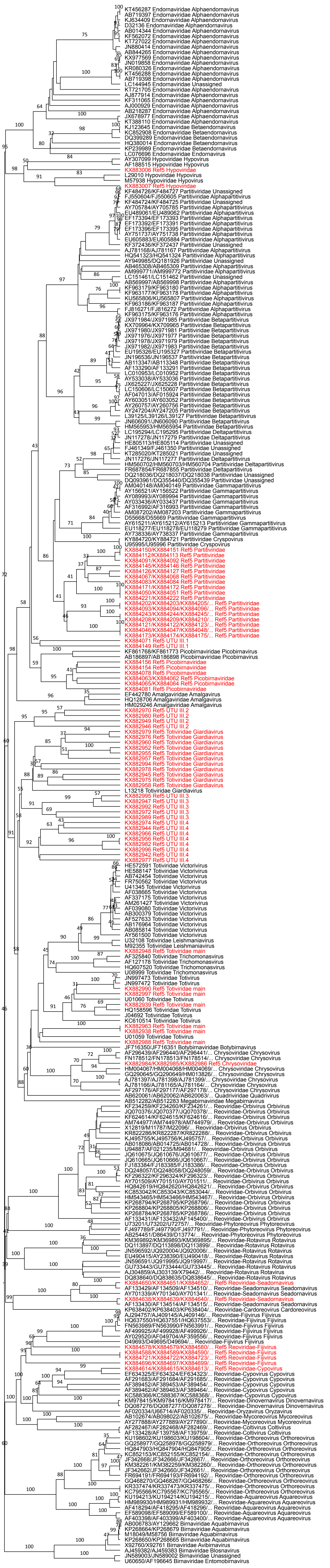

Supplement: Supplementary file 12 — Figures S16–S19. Dendrograms of individual virus sequences of classified and metagenomic viruses in Baltimore groups II, III, IVa and IVb. Full dendrograms that correspond to the collapsed dendrograms shown in Figs. 5, 6, 7, 8 and 9 (lower panels). (ZIP 417 kb) [file 40168_2018_422_MOESM12_ESM.zip › Figure S17.pdf]

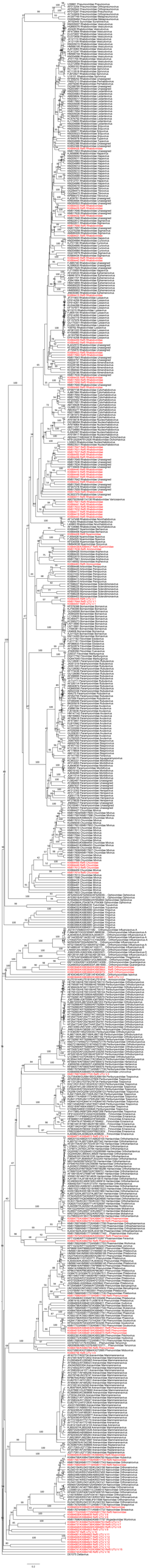

Supplement: Supplementary file 12 — Figures S16–S19. Dendrograms of individual virus sequences of classified and metagenomic viruses in Baltimore groups II, III, IVa and IVb. Full dendrograms that correspond to the collapsed dendrograms shown in Figs. 5, 6, 7, 8 and 9 (lower panels). (ZIP 417 kb) [file 40168_2018_422_MOESM12_ESM.zip › Figure S19.pdf]
