# Supplementary material for: The genomic underpinnings of eukaryotic virus taxonomy: creating a sequence-based framework for family-level virus classification
Source: Microbiome. 2018 Feb 20;6:38. doi: 10.1186/s40168-018-0422-7 (PMC5819261; doi:10.1186/s40168-018-0422-7)
Supplement: Supplementary file 11 — Figures S13–S15. Phylogenetic trees of classified virus groups. Analysis of phylogeny relationships of viruses whose classification by GRAViTy conflicts with their ICTV assignments. (DOCX 825 kb) [file 40168_2018_422_MOESM11_ESM.docx]

FIGURE S13

RdRp PHYLOGENY OF *PICORNAVIRALES* AND RELATED VIRUSES


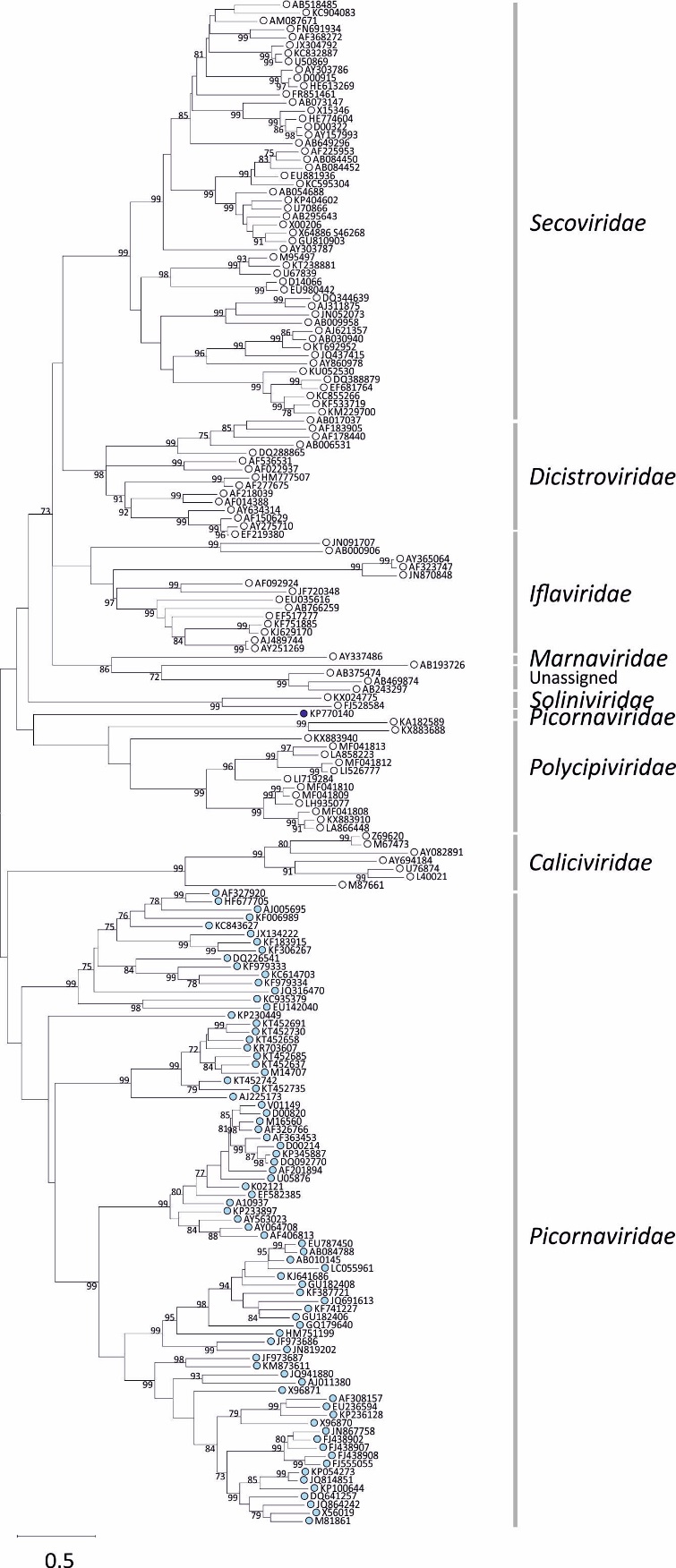


Maximum likelihood tree of the RdRp gene for *Picornavirales* and related viruses using an optimal substitution model (LG + gamma (G) + invariant sites (I)) in the program MEGA6 [1]. Bootstrap re-sampling (100 replicates) was used to indicate robustness of groupings (values ≥70% shown). Paraphyletic members of the *Picornaviridae* are labelled with light blue/blue symbols.

FIGURE S14

RdRp PHYLOGENY OF dsRNA VIRUSES (BALTIMORE GROUP III)


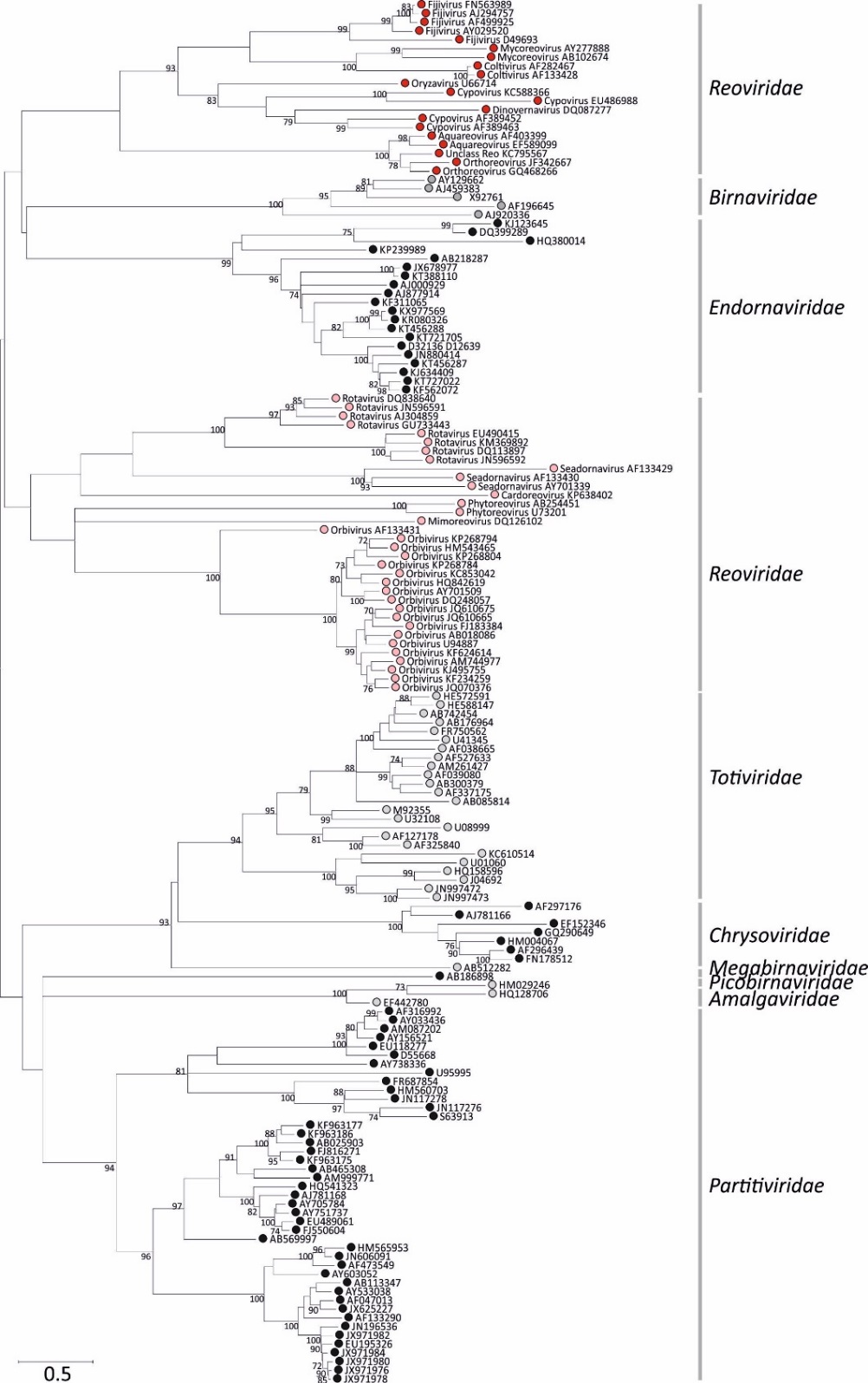


Phylogeny of RdRp gene for Group III viruses by maximum likelihood using an optimal substitution model (LG + G + I) in the program MEGA6 [1]. Bootstrap re-sampling (100 replicates) was used to indicate robustness of groupings (values ≥70% shown). Members of the two sub-families of *Reoviridae* are labelled in red (*Spinareovirinae*) and pink (*Sedoreovirinae*)

FIGURE S15

DNA pol PHYLOGENY OF LARGE DNA VIRUSES (BALTIMORE GROUP I)


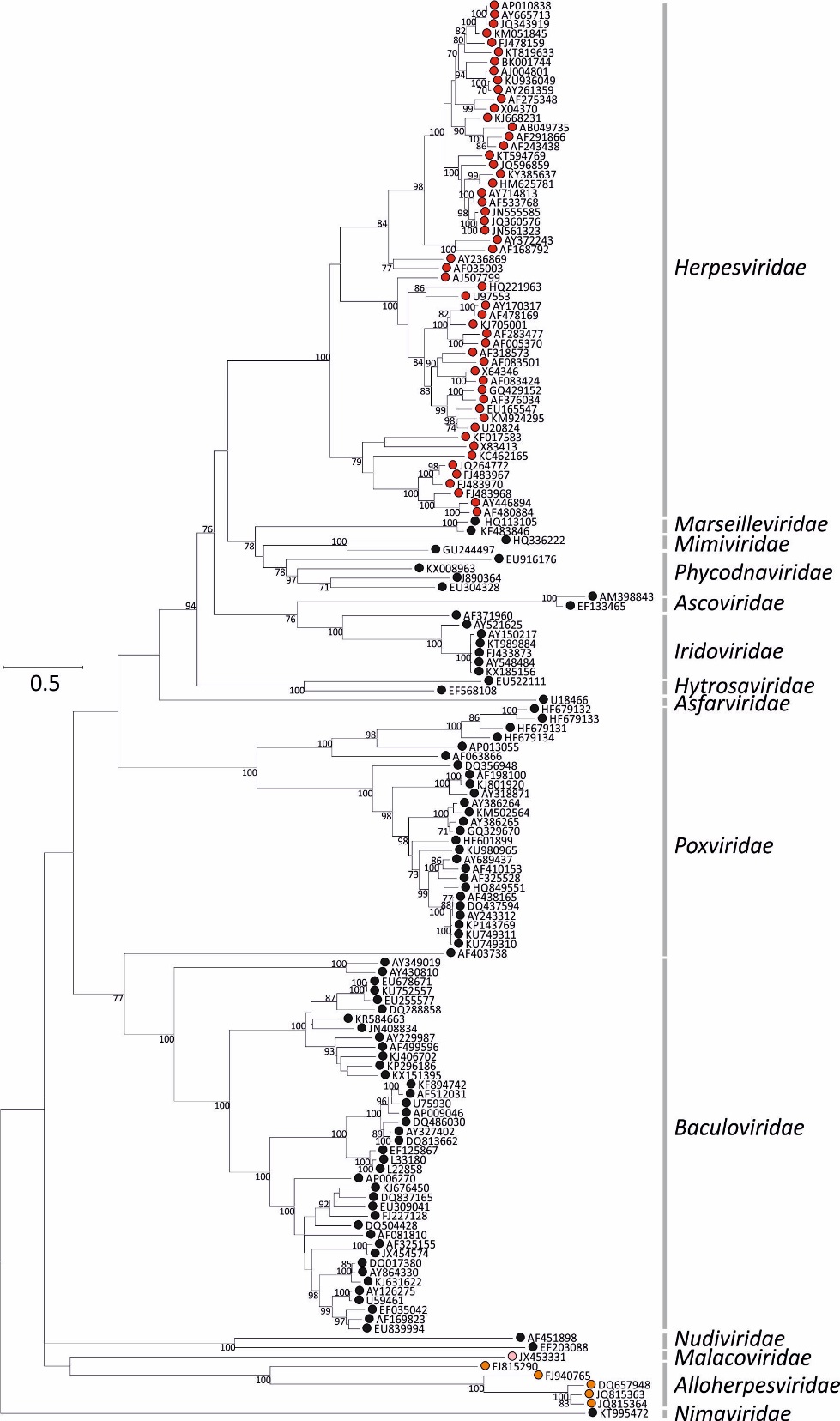


Phylogeny of the DNA polymerase gene for large eukaryotic DNA viruses classified into Group I by maximum likelihood using an optimal substitution model (LG + G + I) in the program MEGA6 [1]. Bootstrap re-sampling (100 replicates) was used to indicate robustness of groupings (values ≥70% shown). Members of the *Herpesvirales* are separately labelled in red, pink and orange.

1. Tamura K, Stecher G, Peterson D, Filipski A, Kumar S: **MEGA6: Molecular Evolutionary Genetics Analysis version 6.0**. *Mol Biol Evol* 2013, **30**(12):2725-2729.
