# Supplementary material for: The genomic underpinnings of eukaryotic virus taxonomy: creating a sequence-based framework for family-level virus classification
Source: Microbiome. 2018 Feb 20;6:38. doi: 10.1186/s40168-018-0422-7 (PMC5819261; doi:10.1186/s40168-018-0422-7)
Supplement: Supplementary file 13 — Analysis of GRAViTy groupings that conflict with ITCV family assignments. Analysis of sequences whose assignment by GRAViTy conflicts with their ICTV classification. (PDF 59 kb) [file 40168_2018_422_MOESM13_ESM.pdf]

SUPPLEMENTARY DATA

Analysis of GRAViTy groupings that conflict with ITCV family assignments

Members of a small number of virus families did not form monophyletic clades on GRAViTy analysis. These outcomes may represent faults in the methodology used for grouping viruses in the current study or alternatively reflects relationships between viruses that were genuinely incongruent with their current ITCV taxonomy assignments. These comprised:

1) Members of the *Luteovirus* genus in the family *Luteoviridae* group with in the family *Tombusviridae*, whereas other luteoviruses form a separate bootstrap supported clade. This reflects the close affinity of the *Luteovirus* replication proteins with those of tombusviruses (1) while capsid and movement proteins resemble those of other members of the *Luteoviridae* and *Sobemoviridae*.

2) One member of the *Sobemoviridae* (Poinsettia latent virus - AJ867490, genus *Polemovirus*) groups within the *Luteovirus* family. This virus is known to be chimaeric, with most of the genome similar in organization and sequence to members of the genus *Polerovirus* (family *Luteoviridae*), whereas the coat protein is most closely related to members of the genus *Sobemovirus*, now assigned to the family *Sobemoviridae*.

3) The resolution of families assigned to the order *Picornavirales* was incomplete. The *Picornaviridae*, through the inclusion of the newly assigned genus, *Ampivirus* (from an amphibian; KP770140) was monophyletic but with relatively low degree of bootstrap support (44%). Some members of the *Iflaviridae* (AF323747, AY365064, KJ186788 and JN870848) were split from the remainder of the family. Similarly, members of the *Spipolycivirus* genus branched separate from other members of the *Polycipiviridae*.

To investigate the genomic basis for these divisions, RdRp sequences of all species assigned to the *Picornavirales* in the five families, along with *Caliciviridae*, *Soliniviridae, Polycipiviridae* and four currently unassigned picorna-like insect virus (AB375474, AB469874, AB243297, AB193726) were aligned and phylogenetic analysis performed (Fig. S13). The resulting tree showed a similar topology to that produced by GRAViTy. There was similarly poor bootstrap support for the grouping of members of the *Picornaviridae* in to a

single lineage and indeed the group was actually paraphyletic with the inclusion of the amphibian virus KP770140.

Members of the *Iflaviridae* were monophyletic but with the same sequences (JN870848, AY365064 and AF323747) that split from the main group on GRAViTy analysis also splitting in the RdRp phylogeny. Similarly, while the *Polycipiviridae* grouped together, the *Spipolycivirus* genus took an outlier position creating a grouping that lacked bootstrap support. Examination of the PPHMMs that linked members of the *Polycipiviridae* revealed that the RdRp was the principal gene that discriminated family members from other virus groups. However, PPHMMs from the structural genes additionally contributed to this separation. As noted previously, however (2), the capsid genes of members of the *Spipolycivirus* genus do not show detectable homology to those of other *Polycipiviridae* (or to any other protein) and this leads to this genus being pushed out of the main family group on GRAViTy analysis.

4) Members of the *Geminiviridae* and *Genomiviridae* did not form bootstrap supported clades and the *Mastrevirus, Eragrovirus, Capulavirus, Becurtovirus* and *Grablovirus* genera of geminiviruses grouped with the genomoviruses, although again without bootstrap support.

5) Rubella virus (Baltimore group IV), classified as the sole member of the genus *Rubivirus* in the family *Togaviridae* grouped separately from other togaviruses. This division is reflected similarly in the RdRp tree contructed from *Tymovirales* and related viruses (Fig. S13). This is expected given their differences in replication mechanisms, morphology and lack of protein sequence similariy on comparisaon with other members of group IV viruses (3).

6) Members of the *Reoviridae* family (Group III) were polyphyletic on GRAViTy analysis (Fig. 1, 2 and Suppl. Data, Figs. S2 and S7). This family is known to possess highly diverse members, currently sub-divided into two sub-families (*Spinareovirinae* and *Sedoreovirinae*) that display distinct morphologies ("turreted" and "smooth" respectively), virion components (single and multiple capsid layers) and genome organisation (4). Using GRAViTy, little (RdRp) or no protein sequence homology is detectable between sub-families, nor between divergent members of the *Sedoreovirinae.* Instead, reoviruses were divided into 4 groups without detectable relatedness between them, comprising (i) *Orbi-, Seadorna-, Cardoreo-*, and *Phytoreovirus* genera (<70% boostrap support); (ii) *Orthoreo-* and *Aquareovirus* (100%

boostrap support); (iii) *Cypo-, Dinoverna-, Oryza-, Fiji-, Mycoreo-*, and *Coltivirus* (100% boostrap support); and (iv) *Rotavirus* (100% boostrap support). In the absence of published analyses of sequence relationships at family level between reoviruses and other dsRNA viruses in Baltimore Group III, an alignment of polymerase gene sequences surrounding the enzymatically active polymerase motif was constructed and phylogeny relationships determined by construction of a maximum likelihood tree (Fig. S14). This analysis split *Reoviridae* into two major clusters, corresponding to their assigned sub-families but without individual bootstrap support. Members of both did however form supported sub-clusters that matched their groupings by PPHMM analysis and degrees of bootstrap support. As previously discussed (5), it is possible that the *Reoviridae* might be split into two or more divisions so that its diversity better matches family assignments of other dsRNA viruses. Our analysis clearly supports such a proposal.

7) The sole member of the *Giadiavirus* genus fails to group with the rest of the *Totiviridae* (which form a bootstrap supported clade elsewhere in the tree). Although Giardia lamblia virus shows structural homology to other totiviruses (6), it differs in host range, ability to infect cells extracellularly and its polymerase gene is phylogenetically distinct, showing no evidence of grouping with RdRps of other totiviruses (Fig. 14).

8) Members of the *Ascoviridae* grouped separately from *Iridoviridae* except the insect virus Diadromus pulcehllus ascovirus 4a (DpAV-4a; CU469068; genus *Toursvirus*), that has an outlier relationship with *Iridoviridae*, on GRAViTy analysis (Figs. 1, 2 and Suppl. Fig. S9). This finding is consistent with the results from previous analysis (7) that reported that DpAV-4a does not belong to *Ascoviridae* and should be moved into either the *Iridoviridae* family or an entirely new family.

9) One member of the *Phycodnaviridae* (AJ890364, genus *Coccolithovirus*) does not group with other members of the family (Figs. 2, and Suppl. Fig. S7). Phycodnaviruses are collectively greatly divergent in sequence, genome organisation and morphology from each other although in this case clearly linked by DNA polymerase gene phylogeny (Fig. S15).

REFERENCES

1. **Miller WA, Liu S, Beckett R.** 2002. Barley yellow dwarf virus: Luteoviridae or Tombusviridae? Mol Plant Pathol **3:**177-183.

2. **Olendraite I, Lukhovitskaya NI, Porter SD, Valles SM, Firth AE.** 2017. Polycipiviridae: a proposed new family of polycistronic picorna-like RNA viruses. J Gen Virol **98:**2368-2378.

3. **Powers A, Huang H, Roehrig J, Strauss E, Weaver S.** 2012. Togaviridae . Ninth Report of the International Committee on Taxonomy of Viruses, p 1103-1110. *In* King AMQ, Adams MJ, Carstens EB, Lefkowitz EJ (ed), Virus Taxonomy: Classification and Nomenclature of Viruses. Elsevier, San Diego.

4. **Attoui H, Mertens PPC, Becnel J, Belaganahalli S, Bergoin M, Brussaard CP, Chappell JD, Ciarlet M, del Vas M, Dermody TS, Dormitzer PR, Duncan R, Fcang Q, Graham R, Guglielmi KM, Harding RM, Hillman B, Makkay A, Marzachì C, Matthijnssens J, Milne RG, Mohd Jaafar F, Mori H, Noordeloos AA, Omura T, Patton JT, Rao S, Maan M, Stoltz D, Suzuki N, Upadhyaya NM, Wei C, Zhou H.** 2012. Reoviridae . Ninth Report of the International Committee on Taxonomy of Viruses, p 541-637. *In* King AMQ, Adams MJ, Carstens EB, Lefkowitz EJ (ed), Virus Taxonomy: Classification and Nomenclature of Viruses. Elsevier, San Diego.

5. **Nibert ML, Duncan R.** 2013. Bioinformatics of recent aqua- and orthoreovirus isolates from fish: evolutionary gain or loss of FAST and fiber proteins and taxonomic implications. PLoS One **8:**e68607.

6. **Janssen ME, Takagi Y, Parent KN, Cardone G, Nibert ML, Baker TS.** 2015. Three-dimensional structure of a protozoal double-stranded RNA virus that infects the enteric pathogen Giardia lamblia. J Virol **89:**1182-1194.

7. **Bigot Y, Renault S, Nicolas J, Moundras C, Demattei MV, Samain S, Bideshi DK, Federici BA.** 2009. Symbiotic virus at the evolutionary intersection of three

types of large DNA viruses; iridoviruses, ascoviruses, and ichnoviruses. PLoS One **4:**e6397.
