# Supplementary material for: The genomic underpinnings of eukaryotic virus taxonomy: creating a sequence-based framework for family-level virus classification
Source: Microbiome. 2018 Feb 20;6:38. doi: 10.1186/s40168-018-0422-7 (PMC5819261; doi:10.1186/s40168-018-0422-7)
Supplement: Supplementary file 10 — Figures S7–S12. Dendrograms of individual virus sequences of classified viruses in Baltimore groups I–V and VI/VII. Full dendrograms that correspond to the collapsed dendrograms shown in Fig. 2. (ZIP 425 kb) [file Figure S11.pdf › Figure S11.pdf]

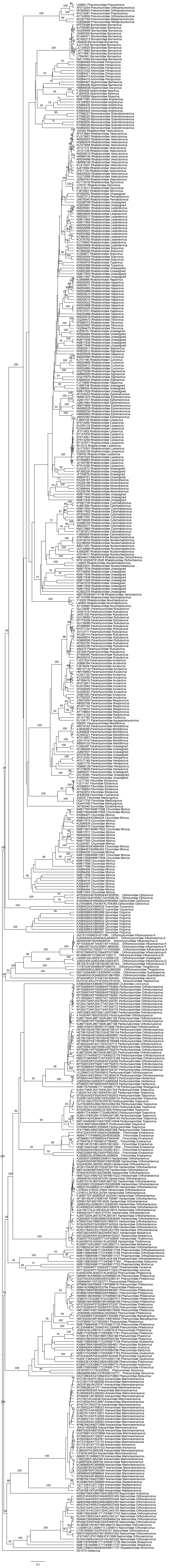
0.2
