# Supplementary material for: The genomic underpinnings of eukaryotic virus taxonomy: creating a sequence-based framework for family-level virus classification
Source: Microbiome. 2018 Feb 20;6:38. doi: 10.1186/s40168-018-0422-7 (PMC5819261; doi:10.1186/s40168-018-0422-7)

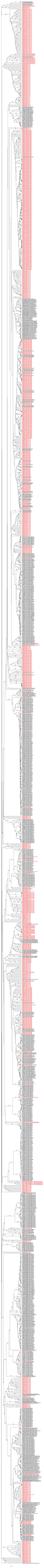

Supplement: Supplementary file 12 — Figures S16–S19. Dendrograms of individual virus sequences of classified and metagenomic viruses in Baltimore groups II, III, IVa and IVb. Full dendrograms that correspond to the collapsed dendrograms shown in Figs. 5, 6, 7, 8 and 9 (lower panels). (ZIP 417 kb) [file Figure S18.pdf › Figure S18.pdf]
